# Perfluorocarbon Nanoemulsions Enhance Therapeutic siRNA Delivery in the Treatment of Pulmonary Fibrosis

**DOI:** 10.1002/advs.202103676

**Published:** 2022-01-07

**Authors:** Ling Ding, Siyuan Tang, Weimin Tang, Deanna D. Mosley, Ao Yu, Diptesh Sil, Svetlana Romanova, Kristina L. Bailey, Daren L. Knoell, Todd A. Wyatt, David Oupický

**Affiliations:** ^1^ Center for Drug Delivery and Nanomedicine Department of Pharmaceutical Sciences College of Pharmacy University of Nebraska Medical Center Omaha NE 68198 USA; ^2^ Department of Internal Medicine Division of Pulmonary and Critical Care and Sleep University of Nebraska Medical Center Omaha NE 68198 USA; ^3^ Department of Pharmacy Practice and Science College of Pharmacy University of Nebraska Medical Center Omaha NE 68198 USA; ^4^ Department of Environmental Agricultural and Occupational Health University of Nebraska Medical Center Omaha NE 68198 USA; ^5^ Research Service Department of Veterans Affairs Omaha‐Western Iowa Health Care System Omaha NE 68105 USA

**Keywords:** CXCR4, idiopathic pulmonary fibrosis, perfluorocarbon emulsion, pulmonary siRNA delivery, STAT3

## Abstract

Local pulmonary administration of therapeutic siRNA represents a promising approach to the treatment of lung fibrosis, which is currently hampered by inefficient delivery. Development of perfluorooctylbromide (PFOB) nanoemulsions as a way of improving the efficiency of pulmonary polycation‐based delivery of siRNA is reported. The results show that the polycation/siRNA/PFOB nanoemulsions are capable of efficiently silencing the expression of STAT3 and inhibiting chemokine receptor CXCR4—two validated targets in pulmonary fibrosis. Both in vitro and in vivo results demonstrate that the nanoemulsions improve mucus penetration and facilitate effective cellular delivery of siRNA. Pulmonary treatment of mice with bleomycin‐induced pulmonary fibrosis shows strong inhibition of the progression of the disease and significant prolongation of animal survival. Overall, the study points to a promising local treatment strategy of pulmonary fibrosis.

## Graphical abstract

This paper reports perfluorocarbon nanoemulsions capable of safe and effective pulmonary siRNA delivery with deep lung penetration and cytosolic siRNA delivery to mediate effective pulmonary STAT3 silencing and CXCR4 inhibition in pulmonary fibrosis. The reported finding may lead to a development of novel treatment of lung fibrosis via noninvasive delivery.

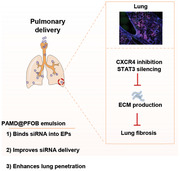

## 1 Introduction

Idiopathic pulmonary fibrosis (IPF) is the most common idiopathic interstitial lung disease. The progression of IPF is categorized by decline of lung function, progressive dyspnea, nonproductive cough, and decreased quality of life.^[^ ^1^ ^]^ Increasing rates of hospital admissions and deaths due to IPF underlie the need for better therapies.^[^ ^1^ ^]^ Development of pulmonary fibrosis is also implicated in severe COVID‐19 infections, further underscoring the urgency.^[^ ^2^ ^]^

Current views of the pathogenesis of IPF rely on repeated subclinical epithelial injury combined with augmented epithelial aging that leads to abnormal repair and the formation of interstitial fibrosis by myofibroblasts.^[^ ^3^ ^]^ The senescence of alveolar epithelial cells and activation and differentiation of myofibroblasts is a central phenotype that promotes IPF.^[^ ^4^ ^]^ Fibroblastic foci, where disordered collections of type II alveolar epithelial cells are present with fibroblasts, are considered a key pathological feature of IPF.^[^ ^5^ ^]^ After decades of clinical trials, there are currently only two FDA approved antifibrotic medications that are considered safe and somewhat effective, nintedanib and pirfenidone.^[^ ^6^ ^]^ The mechanism of pirfenidone is not fully understood, but it possesses both anti‐inflammatory and antifibrotic effects, whereas nintedanib, a tyrosine kinase inhibitor, specifically blocks fibroblast proliferation, migration, differentiation, and the formation of extracellular matrix (ECM).^[^ ^7^ ^]^

With the recent regulatory approvals of the first siRNA therapeutics,^[^ ^8^ ^]^ the development of additional siRNA‐based medicines for a broad range of diseases has become increasingly feasible. Direct delivery of siRNA into the lungs has beneficial characteristics, such as avoidance of undesired serum interactions and degradation. However, the anatomy of the respiratory tract is complex and creates obstacles for effective delivery. Several preclinical studies in pulmonary fibrosis demonstrated efficacy using siRNA formulations aimed at silencing the expression of collagen‐specific chaperone heat shock protein 47 (HSP47),^[^ ^9^ ^]^ Janus kinase type 2 (JAK 2),^[^ ^10^ ^]^ E1A binding protein P300 (EP300),^[^ ^11^ ^]^ plasminogen activator inhibitor‐1 (PAI‐1),^[^ ^12^ ^]^ and NLR family pyrin domain containing 3 (NLRP3).^[^ ^13^ ^]^ Related to this, two human clinical trials have used siRNA inhalation strategies to treat respiratory syncytial virus (RSV) (ALN‐RSV01)^[^ ^14^ ^]^ and asthma (Excellair) via Syk silencing.^[^ ^15^ ^]^

Perfluorocarbon (PFC) nanoemulsions are versatile soft nanomaterials with a unique set of properties suitable for multiple biomedical applications, including drug delivery and bioimaging. Unlike self‐assembled soft materials like micelles, PFC nanoemulsions are kinetically stable systems where the PFC oil phase is stabilized with amphiphilic surfactants.^[^ ^16^ ^]^ Nanoemulsions have a potentially large loading capacity for active molecules, with release properties controlled by the nature of the oil phase and choice of surfactant. PFC nanoemulsions also have high oxygen solubility, which prompted their first development as blood substitutes. Perfluorooctylbromide (PFOB) is among the most commonly used PFCs in biomedical applications.^[^ ^17^ ^]^ PFOB nanoemulsions have been studied in the treatment of cancer and atherosclerosis.^[^ ^18^ ^]^ Pulmonary administration of PLGA‐PEG/PFOB nanoemulsions have been shown to improve lung ventilation.^[^ ^19^ ^]^ Photodynamic therapy has been another successful application of PFOB emulsions, which benefits from high oxygen solubility.^[^ ^17^, ^20^ ^]^

Chronic dysfunctional inflammatory response and aberrant self‐repair are critical factors in progressive IPF. This involves the recruitment of immune and mesenchymal cells by chemokines and chemokine receptors, which play a critical role in cell migration and also serve as potential therapeutic targets.^[^ ^21^ ^]^ Invasion of fibroblasts into the alveolar region results in collagen deposition and fibroblast differentiation into myofibroblasts.^[^ ^22^ ^]^ The G‐protein coupled C‐X‐C chemokine receptor type 4 (CXCR4) is a candidate therapeutic target in IPF because of its role in the recruitment of CXCR4‐positive fibrocytes to the lung that increase the extent of fibrosis.^[^ ^23^ ^]^ Stromal cell‐derived factor‐1 (SDF‐1) is a CXCR4 ligand generally secreted by bone marrow stroma cells. However, SDF‐1 also can be secreted by pulmonary fibroblasts and alveolar epithelial type II cells (AEC II), leading to activation and recruitment of CXCR4‐ positive cells.^[^ ^24^ ^]^ CXCR4 is abundantly expressed in IPF patients, with prominent expression in honeycomb cysts and the distal airway epithelium.^[^ ^25^ ^]^ CXCR4 inhibition attenuates the progression of bleomycin (BLM)‐induced pulmonary fibrosis in mice.^[^ ^12a^ ^]^

**Figure 1:**
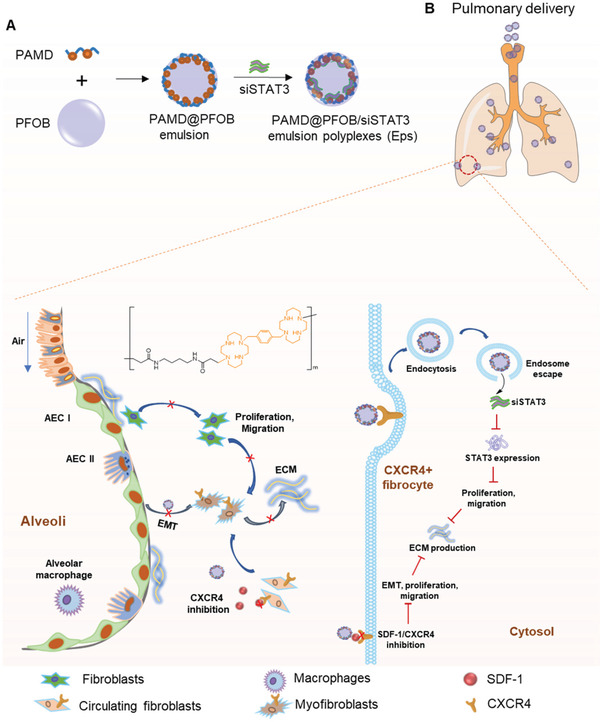
Preparation and proposed mechanism of action of PAMD@PFOB/siSTAT3 emulsion polyplexes (EPs) in inhibiting pulmonary fibrosis. A) Preparation of PAMD@PFOB/siSTAT3 EPs. PFOB emulsions are stabilized with PAMD to obtain the PAMD@PFOB emulsion. PAMD@PFOB/siSTAT3 is then formed by electrostatic adsorption of siRNA to the emulsion. B) PAMD@PFOB/siSTAT3 is administrated by pulmonary delivery and modulate the pulmonary fibrosis by inhibiting EMT and fibroblasts proliferation and migration, then decreasing the ECM production.

Excessive phosphorylation of a signal transducer and activator of transcription 3 (STAT3) has been implicated as a driver of aberrant fibroblast activation.^[^ ^26^ ^]^ STAT3 is a cytoplasmic transcription factor with important role in cell proliferation, migration, differentiation, and survival.^[^ ^27^ ^]^ TGF‐*β* signaling triggers phosphorylation of JAK2, which then interacts with and phosphorylates STAT3 to induce a fibrotic response.^[^ ^28^ ^]^ STAT3 signaling is hyperactivated in systemic sclerosis in a TGF‐*β*‐dependent manner, suggesting that STAT3 may be a core mediator of fibrosis. In this study, we report on the development of PFC nanoemulsions, termed emulsion polyplexes (EPs), for pulmonary siRNA delivery that combine CXCR4 inhibition and STAT3 gene silencing. We present results demonstrating in vitro antifibrotic activity, favorable pulmonary biodistribution, and the potential to reduce the severity and prolong survival in BLM‐induced lung fibrosis (**Figure** **1**).

## 2 Results and Discussion

### 2.1 Physiochemical Characterization of PAMD@PFOB/siRNA EPs

**Figure 2:**
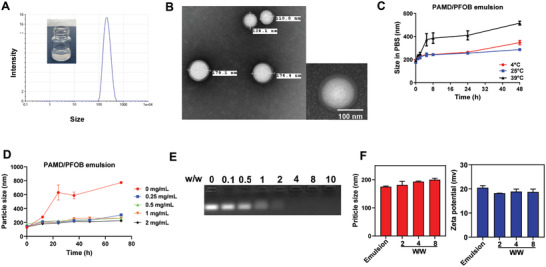
Characterization of PAMD@PFOB emulsion and PAMD@PFOB/siRNA emulsion polyplexes (EPs). A) Particle size, B) representative TEM images, and C) colloidal stability of the PAMD@PFOB emulsion. D) The stability of the PAMD@PFOB emulsion stabilized with different PAMD concentrations. E) siRNA binding ability of PAMD@PFOB emulsion by agarose gel retardation assay. F) Hydrodynamic particle size and zeta potential of the EPs at different PAMD/siRNA w/w ratios.

We have developed CXCR4‐inhibiting polycations, named PAMD, that form nanosized polyplexes with nucleic acids, such as siRNA, miRNA, and DNA.^[^ ^29^ ^]^ In this study, we have used PAMD as a surfactant to stabilize PFOB nanoemulsions and to form EPs to improve the pulmonary siRNA delivery in mice with pulmonary fibrosis. We synthesized PAMD with a weight‐average molecular weight of 10 kDa and ^1^H‐NMR spectrum shown in Figure S1 (Supporting Information). PAMD@PFOB (o/w) primary emulsions were prepared via a one‐step ultrasonication method^[^ ^17a^ ^]^ using 1% v/v PFOB in the presence of different concentrations of PAMD (**Figure** **2**A). The core–shell structure of the emulsions consisted of the PFOB core, which was stabilized by a layer of PAMD shell (Figure 2B). This specific structure enabled exposure of PAMD and its CXCR4‐binding moieties on the emulsion surface while providing the positive surface charge necessary for siRNA binding. The PAMD@PFOB emulsions remained relatively stable for at least three days at room temperature as indicated by a particle size increase of less than 20% (Figure 2C). As shown in Figure 2D, even the lowest tested concentration of PAMD (0.25 mg mL^−1^) was sufficient to stabilize the 1% v/v PFOB emulsion and limit particle size growth. The particle size of the PAMD@PFOB emulsion prepared with 2 mg mL^−1^ PAMD was 175 ± 2 nm and the zeta potential was 20.5 ± 0.9 mV (Figure 2A,F). The ability of siRNA to bind to the surface of the PAMD@PFOB emulsions was then evaluated by a gel retardation assay (Figure 2E). The EPs were prepared by mixing increasing amounts of PAMD@PFOB emulsion with siRNA solution. We found that siRNA bound completely to the emulsion at a w/w ratio of PAMD/siRNA (excluding PFOB) above 2. We used w/w = 4 in subsequent in vitro and in vivo studies to maintain slight excess of CXCR4‐binding moieties not engaged in electrostatic interactions with siRNA. The size of EPs that were prepared at w/w = 4 was ≈190 nm with zeta potential ≈19 mV (Figure 2F). The residual positive zeta potential confirmed that some CXCR4 binding moieties remained presented on the surface of the emulsion despite siRNA binding. CXCR4 redistribution assay was used to evaluate the CXCR4 antagonism of PAMD@PFOB emulsion (Figure S2, Supporting Information). Both PAMD and PAMD@PFOB displayed dose‐dependent CXCR4 antagonism.

### 2.2 Cytosolic siRNA Delivery and Antifibrotic Effect of EPs in Primary Mouse and Human Lung Fibroblasts

**Figure 3:**
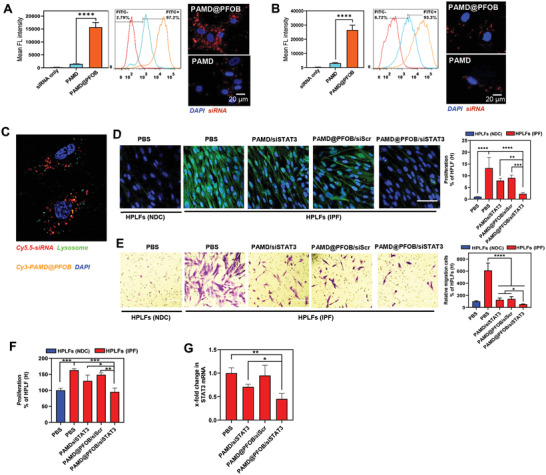
Effect of CXCR4 inhibition and STAT3 gene silencing on fibroblast proliferation and migration. Cytosolic siRNA delivery in A) HPLFs (IPF), B) MPLFs (IPF), scale bar = 20 µm. C) Endosome escape of PAMD@PFOB/siRNA. D) Immunofluorescence staining of *α*‐SMA (green). Cell nuclei stained with DAPI (blue), scale bar = 100 µm (*n* = 6, mean ± SD, one‐way ANOVA with Tukey's multiple comparisons test). E) Representative images of fibroblast migration with different treatments (*n* = 3, mean ± SD, one‐way ANOVA with Tukey's multiple comparisons test). F) Fibroblast proliferation following different treatments (*n* = 3, mean ± SD, one‐way ANOVA with Tukey's multiple comparisons test). G) STAT3 mRNA expression in the IPF HPLFs after treatment (*n* = 3, mean ± SD, one‐way ANOVA with Tukey's multiple comparisons test). Significance was indicated as *p <0 .05, **p < 0.01, ***p < 0.001, and ****p < 0.0001.

We first assessed the toxicity of EPs in mouse primary lung fibroblasts (MPLFs) that were isolated from lungs with established fibrosis [MPLFs (IPF)], as well as human primary lung fibroblasts (HPLFs) isolated from IPF patients [HPLFs (IPF)], and non‐disease control [HPLFs (NDC)] (Figure S3, Supporting Information). MPLFs (IPF) exhibited less cytotoxicity in response to PAMD and EPs than both NDC and IPF HPLFs. No significant cytotoxicity was observed with the EP formulations used at a w/w ratio of 4 and 100 × 10^−9^ m siRNA and this formulation was thus used in all subsequent studies. Based on prior studies, we hypothesized that the localization of the PAMD and siRNA on the surface of the PFOB nanoemulsions would improve siRNA cytosolic delivery. EPs were prepared with fluorescently labeled siRNA and cell internalization in the HPLFs (IPF, **Figure** **3**A) and MPLFs (IPF, Figure 3B) was examined by confocal microscopy and flow cytometry. We found that PAMD@PFOB/siRNA EPs significantly improved siRNA cellular uptake when compared with the PAMD/siRNA in both the human primary and mouse primary fibroblasts obtained from fibrotic lungs. This result was also confirmed by flow cytometry. Endosomal escape of the PAMD@PFOB/siRNA EPs in HPLF (IPF) was evaluated using LysoView to stain lysosomes. After 4 h incubation, Cy5.5‐siRNA was found mostly in the cytoplasm with only a small amount present in the lysosomes as suggested by the limited overlap with the LysoView signal (Figure 3C). These results suggested that PFOB nanoemulsion can enhance the cytosolic siRNA delivery. However, the mechanism governing this process is poorly understood. The cellular uptake and endosome escape results showed that most siRNA could escape from the lysosomes and be transported to the cytosol, which suggested that the mechanism may involve the perturbation of intracellular phospholipid membranes.^[^ ^30^ ^]^ Further studies need to be performed to gain better understanding.

Differentiated fibroblasts play a key role in the progression of IPF and strategies that modulate their function may have a significant impact on the treatment of IPF.^[^ ^31^ ^]^ The characteristic differential expression of cytoskeletal proteins such as *α* smooth muscle actin (*α*‐SMA) can be used as a marker of activated lung myofibroblasts. We evaluated expression of *α*‐SMA in IPF and NDC HPLFs and determined the effect of treatment with EPs (Figure 3D). As expected, the IPF HPLFs showed higher expression of α‐SMA than HPLFs (NDC), and treatment with PAMD@PFOB/siSTAT3 EPs significantly decreased the expression. Control treatment with EPs prepared with scrambled siRNA and polyplexes without the PFOB emulsion showed a less pronounced effect on *α*‐SMA expression. Fibroblasts in IPF lungs represent a population of cells with diverse phenotypes and functions. The accumulation of fibroblasts and myofibroblasts is a hallmark of IPF, leading to the production of excessive ECM.^[^ ^32^ ^]^ Activated myofibroblasts can induce apoptosis of epithelial cells, which contributes to the absence of re‐epithelialization and thereby perpetuating IPF.^[^ ^33^ ^]^ Activated fibroblasts from IPF can be also distinguished by increased migration as confirmed in Figure S4A (Supporting Information) when compared with NDC HPLFs. CXCR4, STAT3, col1a1, and col1a2 expression were significantly elevated in IPF HPLFs when compared with NDC HPLFs (Figure S4B,C, Supporting Information). Importantly, when treating the IPF HPLFs with the PAMD@PFOB/siSTAT3 EPs, the expression of STAT3, col1a1, and col1a2 decreased and reached the levels similar to the control HPLF (NDC) (Figure S4C, Supporting Information). We also examined the role of CXCR4 and STAT3 inhibition in fibroblast proliferation and migration (Figure 3E,F). IPF HPLFs were treated with PAMD/siSTAT3, PAMD@PFOB/siScr, and PAMD@PFOB/siSTAT3. All the used treatments were capable of CXCR4 inhibition and achieved nearly complete inhibition of cell migration, similar to NDC HPLFs. Moreover, treating the HPLFs (IPF) with PAMD@PFOB/siSTAT3 resulted in better migration inhibition than treatments with PAMD/siSTAT3 and PAMD@PFOB/siScr. Fibroblast proliferation experiments showed similar results in that the IPF HPLFs displayed significantly increased proliferation (Figure 3F). To further confirm that proliferation of fibroblasts requires CXCR4 and STAT3 activation, we treated cells with PAMD@PFOB/siSTAT3 EPs. As shown in Figure 3F, PAMD@PFOB/siSTAT3 EPs significantly suppressed fibroblasts proliferation. We also evaluated the STAT3 gene silencing efficacy in the HPLFs IPF. Uptake of siRNA was improved significantly by the EPs and resulted in improved STAT3 gene silencing efficacy (Figure 3G).

Importantly, our data showed that CXCR4, STAT3, col1a1, and col1a2 were significantly overexpressed in IPF HPLFs compared with NDC (Figure S4, Supporting Information). Treating IPF HPLFs with PAMD@PFOB/siSTAT3 EPs significantly decreased both proliferation and migration. CXCR4 and STAT3 signaling contribute to the differentiation of fibroblasts into myofibroblasts as well as the proliferation and migration of fibroblasts. This is important because interstitial lung fibroblasts proliferate and migrate to the injury site where they are responsible for fibroblast proliferation and transformation to myofibroblasts, as well as synthesis and deposition of ECM.^[^ ^7^ ^]^ In the present study, STAT3 inhibition prevented fibroblast proliferation and migration, thus decreasing the production of ECM and attenuating the progression of pulmonary fibrosis. Taken together, these results with primary human cells provide initial in vitro evidence for antifibrotic effects by EPs that combine CXCR4 antagonism with STAT3 gene silencing.

### 2.3 Enhanced Mucus Stability and Penetration and Decreased Ciliary Beat Frequency (CBF) by EPs

Pulmonary delivery of siRNA is a complex process that demands sufficient stability of the delivery system to reach the desired location in the lungs. The mucus layer and pulmonary surfactant that cover the airway epithelium are major obstacles to pulmonary siRNA delivery, in addition to other host defense factors that capture the particles and eliminate them from the lungs.^[^ ^34^ ^]^

As part of our comprehensive stability testing, resistance against polyelectrolyte exchange causing the release of siRNA from EPs was evaluated by incubation with a natural polyanion heparin. As shown in Figure S5 (Supporting Information), siRNA was completely released from the EPs when the heparin concentration reached above 80 µg mL^−1^ which indicated to us satisfactory stability.

**Figure 4:**
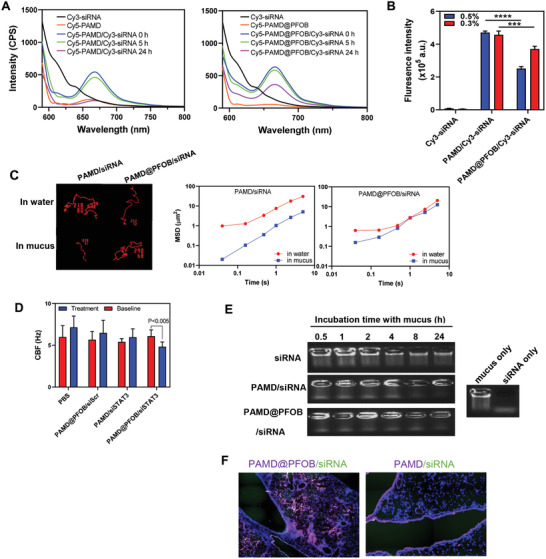
PFOB improved the mucus penetration of PAMD@PFOB/siRNA EPs and PAMD@PFOB/siSTAT3 EPs inhibited the ciliary beat frequency (CBF). A) Fluorescence emission spectra after coincubation with 10% COPD mucus for 0, 5, and 24 h. B) The fluorescence intensity of the aggregates following 5 h coincubation (*n* = 4, mean ± SD, Student's *t*‐test). C) Representative trajectories in the mucus and water during 20 s movies. MSD as a function of time. D) PAMD@PFOB/siSTAT3 EPs inhibit the CBF (*n* = 3, mean ± SD, Student's *t*‐test). E) Mucus stability assays of PAMD@PFOB/siRNA EPs compared with free siRNA and PAMD/siRNA. All samples were incubated with 80 µg mL^−1^ heparin for 30 min immediately prior the electrophoresis to release siRNA from the particles. F) Distribution of PAMD/siRNA and PAMD@PFOB/siRNA EPs in lung epithelial tissue following the intratracheal administration 5 h after the treatment in mice with established pulmonary fibrosis. Significance was indicated as *p < 0.05, **p < 0.01, ***p < 0.001, and ****p < 0.0001.

Stability and penetration of the fluorescently labeled nanoemulsions (Cy5‐PAMD/Cy3‐siRNA) were probed in mucus obtained from patients with chronic obstructive pulmonary disease (COPD) using fluorescence resonance energy transfer (FRET) by labeling separate batches of EPs with Cy3‐siRNA and Cy5‐PAMD (a known FRET couple).^[^ ^35^ ^]^ As shown in **Figure** **4**A, after 24 h of coincubation with mucus, a significant decrease in FRET signal (fluorescence intensity at 667 nm upon excitation at 550 nm) was exclusively observed for the PAMD‐Cy5/Cy3‐siRNA group, suggesting disassembly of the polyplexes. After 24 h in 10% mucus, the EPs were more stable, whereas the FRET changes in case of polyplexes (PAMD‐Cy5/Cy3‐siRNA) suggested that there were almost no remaining particles at 24 h. Thus, the PAMD@PFOB/siRNA EPs remained more stable in mucus and prevented undesired dissociation and siRNA release. Aggregation in mucus may also contribute to poor delivery and limit efficacy of the particles. Therefore, adsorption of mucin to EPs was investigated by quantitating the amount of EP/mucin aggregates (Figure 4B). In this assay, higher fluorescence intensity corresponds to increased formation of the particle/mucin aggregates. After 5 h in mucus, the fluorescence intensity of the PAMD/Cy3‐siRNA group was higher than the PAMD@PFOB/Cy3‐siRNA EPs group, suggesting more of the polyplex/mucin aggregation than the EPs/mucin, proving that the EPs aggregated significantly less than the control PAMD/siRNA polyplexes both in 0.3% and 0.5% mucus.

Mucus penetration properties of PAMD@PFOB/siRNA EPs were evaluated using NanoSight to track the trajectories of the nanoparticles in mucus. As shown in Figure 4C, the movement of PAMD/siRNA polyplexes in the mucus was severely impeded when compared with the movement in water. However, the movement of PAMD@PFOB/siRNA EPs in the mucus remained fast. Importantly, the geometric averaged mean square displacement (MSD) values for PAMD@PFOB/siRNA also remained relatively stable when compared with the MSD values for PAMD@PFOB/siRNA in water. In contrast, the MSD of PAMD/siRNA decreased significantly when compared with the water. Both mucus and pulmonary surfactant are important in defining the fate of the materials in the lung. We thus evaluated the effect of porcine pulmonary surfactant (Curosurf) on the stability of PAMD@PFOB/siRNA EPs (Figure S6, Supporting Information). Using FRET, agarose gel, and TEM showed that PAMD@PFOB/siRNA EPs retained their integrity and remained stable in the presence of pulmonary surfactant.

We evaluated the influence of EPs treatment on CBF. The cilia were assayed by measuring the average number of motile points in multiple whole field measurements of the mouse tracheal epithelial cells (MTEC) grown at an air–liquid interface (ALI) (Figure 4D). Treating the MTEC with PBS did not significantly change CBF. In contrast, there was a decrease in CBF compared to the PBS group when MTEC were treated with PAMD@PFOB/siSTAT3. Because particle stimulation of cilia is a well‐established concept,^[^ ^36^ ^]^ this result may point to benefits for the inhalation of EPs as it may decrease the extent of their clearance. The mechanism of PAMD@PFOB/siSTAT3 influence on the CBF in MTEC may involve silencing STAT3 expression but this requires further investigation.

The siRNA stability in the EPs in mucus was also evaluated via agarose gel electrophoresis (Figure 4E). Compared with free siRNA and PAMD/siRNA, PAMD@PFOB/siRNA EPs were better at protecting siRNA from mucus‐induced degradation. Moreover, in agreement with the above in vitro studies that demonstrated enhanced mucus and pulmonary surfactant stability and decreased CBFs by EPs, the histological results showed that the distribution of PAMD/siRNA polyplexes was confined to the mucus layer of the airways with extremely limited penetration to the deeper areas of the lung (Figure 4F). In contrast, the EPs could efficiently permeate through the mucus layer and penetrate into deeper regions of the lung. We contend that unlike most PFCs, PFOBs has less lipophilicity due to the bromine atom in its structure.^[^ ^37^ ^]^ This may in turn decrease the interaction between EPs and negatively charged mucin glycoproteins, which may help with mucus penetration.

### 2.4 Histology Analysis of the Lungs from IPF Patients and Mice with Pulmonary Fibrosis

**Figure 5:**
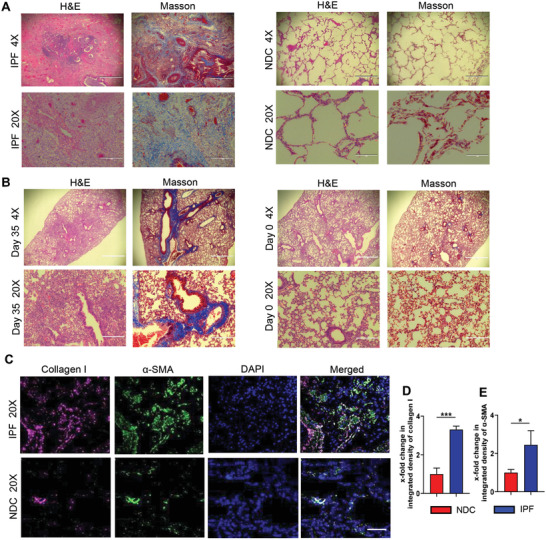
Histology analysis of the lungs from IPF patients compared with NDC, and BLM‐induced pulmonary fibrosis in mice (Day 35) compared with healthy (Day 0) control. A) H&E and Masson staining of the human samples. (IPF patients compared with the NDC). B) H&E and Masson staining of lungs from BLM‐induced fibrosis (Day 35) compared with healthy (Day 0) control. C) Representative images of immunofluorescence staining for collagen I (red) and *α*‐SMA (green) co‐stained with DAPI (blue). D) Quantitative analysis of immunofluorescence staining for collagen I (*n* = 3, mean ± SD, Student's *t*‐test). E) Quantitative analysis of immunofluorescence staining for *α*‐SMA (*n* = 3, mean ± SD, Student's *t*‐test). 4×: Scale bar = 1000 µm. 20×: scale bar = 200 µm. Significance was indicated as *p < 0.05, **p < 0.01, and ***p < 0.001.

**Figure** **5**A,B shows H&E and Masson's trichrome stained slides from IPF patients and mice with BLM‐induced pulmonary fibrosis, together with healthy controls. Healthy lung (NDC) appeared normal with intact alveolar size and structure (Figure 5A). As expected, samples from the IPF patients showed dense thickening of the lung parenchyma, displacement and damage to the alveolar walls, and evident perivascular fibrosis. Similar results were also seen in the mice with BLM‐induced pulmonary fibrosis, validating that the animal model used in this study resembles the human disease (Figure 5B). Masson's trichrome staining also showed large amounts of collagen deposition in the IPF patient lungs compared to NDC, as confirmed by increased immunostaining of collagen I and *α*‐SMA (Figure 5C–E). Collagen I (red) and *α*‐SMA (green) staining was significantly more abundant in IPF than in the NDC lung sections.

**Figure 6:**
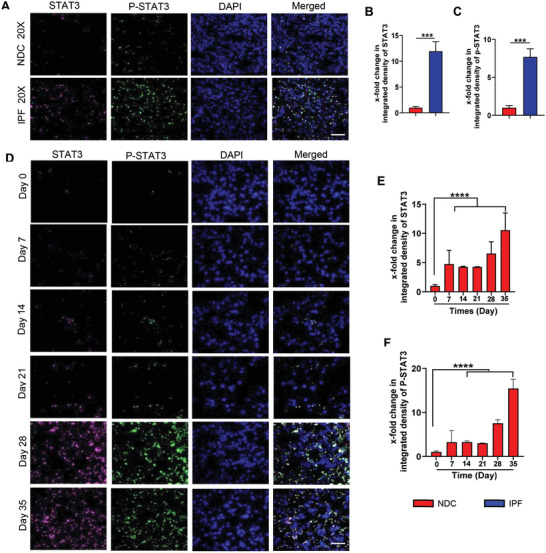
Activation of STAT3 signaling in the lungs of IPF patients and in BLM‐induced pulmonary fibrosis in mice. A) Representative images of immunofluorescence staining for P‐STAT3 (green) and total STAT3 (red) co‐stained with DAPI (blue) in IPF patient and NDC samples. Scale bar = 200 µm. B) Quantitative analysis of immunofluorescence staining for (*n* = 3, mean ± SD, Student's *t*‐test). C) Quantitative analysis of immunofluorescence staining for P‐STAT3 (*n* = 3, mean ± SD, Student's *t*‐test). D) Representative images of immunofluorescence staining for P‐STAT3 (green) and total STAT3 (red) co‐stained with DAPI (blue) in different stages of pulmonary fibrosis in the BLM mouse model. Scale bar = 100 µm. Quantitative analysis of immunofluorescence staining for E) STAT3 and F) P‐STAT3 (*n* = 3, mean ± SD, one‐way ANOVA with Tukey's multiple comparisons test). Significance was indicated as *p < 0.05, **p < 0.01, ***p < 0.001, and ****p < 0.0001.

STAT3 expression and activation were analyzed by quantifying the extent of phosphorylation at tyrosine 705 (p‐STAT3), a marker for STAT3 activation. Consistent with *α*‐SMA and collagen I expression, increased p‐STAT3 and total STAT3 were observed in the parenchyma of IPF patient samples (*P* < 0.0005 vs NDC), whereas only a few cells were positive for p‐STAT3 in healthy NDC (**Figure** **6**A–C). The activation of STAT3 signaling in mice with pulmonary fibrosis was also evaluated in experimental models of BLM‐induced pulmonary fibrosis (Figure 6D–F). The fibrotic mouse lungs showed significantly increased p‐STAT3 and total STAT3 as compared to control healthy mice. Increased STAT3 signaling was detected after four injections of BLM at two weeks after first injection and it persisted throughout the transition to established fibrosis.

### 2.5 Improved Pulmonary Delivery of siRNA by EPs

**Figure 7:**
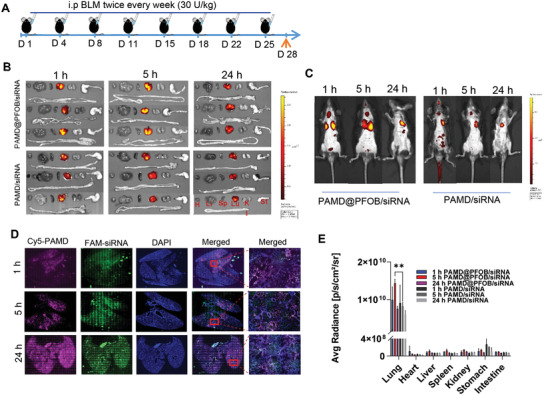
Biodistribution of EPs in BLM‐induced pulmonary fibrosis. A) Timeline of the experiment. B) Ex vivo fluorescence imaging of major organs after intratracheal instillation of Cy5‐PAMD@PFOB/FAM‐siRNA EPs and Cy5‐PAMD/FAM‐siRNA polyplexes. C) Whole‐body fluorescence imaging. D) Intra‐lung distribution of the PAMD@PFOB/siRNA EPs prepared with Cy5‐labeled PAMD@PFOB (red), FAM‐siRNA (green), and DAPI (blue). E) Ex vivo quantification of fluorescence distribution in major organs at different time points (*n* = 3, mean ± SD, Student's *t*‐test). Significance was indicated as *p < 0.05, **p < 0.01, and ***p < 0.001.

We used a lung fibrosis model based on repeated intraperitoneal injection of BLM in C57BL/6 mice (**Figure** **7**A). This model mimics human disease with similar lung pathology, including injury and activation of epithelial cells and inflammatory cell infiltrates, the production of proliferation factors, collagen deposition, and parenchymal consolidation.^[^ ^38^ ^]^ The model progresses through the following three stages: 1) acute injury and inflammation phase (day 1–12) that is characterized by an influx of inflammatory cells and upregulation of proinflammatory cytokines and chemokines; 2) the transition phase from inflammation to active fibrosis (day 12–24), which shows gradual subsiding of the inflammatory response with an accompanying increase in fibroproliferation, myofibroblast emergence, and increase in the expression of *α*‐SMA, collagen I, CXCR4, STAT3, and p‐STAT3 (Figures S7 and S8, Supporting Information); and 3) the established chronic fibrosis stage (day 25–42), which is characterized by the expansion of the myofibroblast population and substantial deposition of ECM.

We first evaluated the biodistribution of EPs at the different stages of the BLM‐induced pulmonary fibrosis (Figure S9, Supporting Information). Using dual‐labeled EPs (Cy5‐PAMD@PFOB/FAM‐siRNA) that were instilled intratracheally (40 µL, 15 µg siRNA per mouse), the biodistribution was determined 5 h post‐instillation using whole‐body imaging and ex vivo imaging of excised lungs and other major organs. The results showed strong EP accumulation during the second stage of fibrosis when the transition from inflammation to active fibrosis occurs. The EP lung clearance in healthy mice was faster than in mice with established pulmonary fibrosis (Figure S9B–D, Supporting Information). Importantly, EPs penetrated throughout the entire lung even at stage 3, where extensive collagen and ECM deposition were present (Figure S9E, Supporting Information).

Lung deposition and penetration were further evaluated in detail in mice with established fibrosis (Figure 7A). Cy5‐PAMD@PFOB/FAM‐siRNA (40 µL, w/w = 4, siRNA 15 µg per mice) or Cy5‐PAMD/FAM‐siRNA polyplexes were intratracheally instilled and animals were sacrificed at 1, 5, and 24 h, followed by fluorescence imaging (Figure 7B,C). At 5 h, EPs showed the highest lung accumulation (Figure 7E), most likely due to better mucus and pulmonary surfactant stability and penetration of EPs than the simple polyplexes without PFOB. The Cy5‐PAMD/FAM‐siRNA polyplexes were mostly trapped in the mucus barrier and pulmonary surfactant layer and subject to faster clearance. The fluorescent signal remained localized in the lung and not found in the other organs within 24 h post‐instillation.

To further explore the extent of penetration into the deep lung, the intra‐lung distribution of labeled EPs was evaluated via confocal microscopy. Lungs were collected, inflated and fixed with OCT, and embedded for frozen tissue sectioning (Figure 7D). Both Cy5‐PAMD (red) and FAM‐siRNA (green) fluorescence colocalized in the lungs, suggesting that the EPs integrity was retained during the delivery phase. The intra‐lung distribution of labeled PAMD/siRNA showed that most polyplexes were trapped in the bronchus even 24 h post‐administration (Figure S10, Supporting Information). We were also able to demonstrate that EPs penetrated into lower lobes as early as 1 h post administration and spread to all lobes within 5 h.

### 2.6 Therapeutic Efficacy of EPs in BLM‐Induced Pulmonary Fibrosis Mice

**Figure 8:**
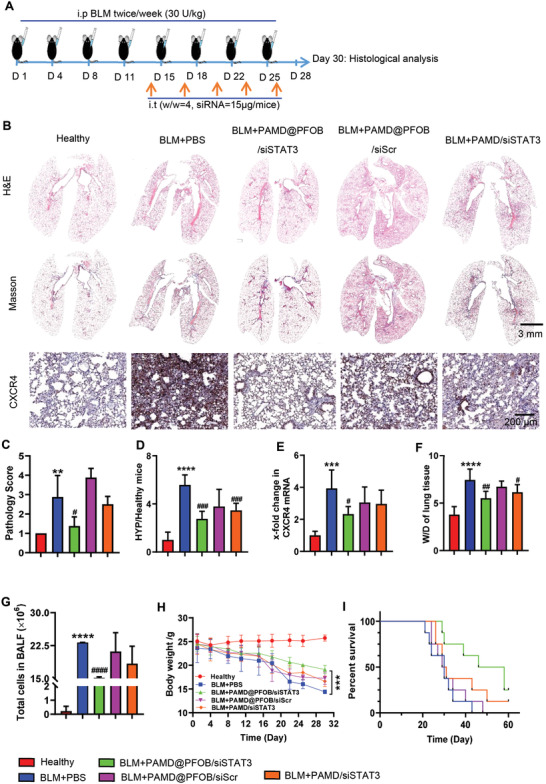
Therapeutic activity of EPs in BLM‐induced pulmonary fibrosis. A) Treatment timeline. B) Representative images of lung sections stained with H&E, Masson's trichrome, and CXCR4 immunochemistry staining. C) Pathology scores of the lungs (*n* = 4, mean ± SD, one‐way ANOVA with Tukey's multiple comparisons test). D) Hydroxyproline levels in the lungs (*n* = 6, mean ± SD, one‐way ANOVA with Tukey's multiple comparisons test). E) The mRNA level of CXCR4 (*n* = 6, mean ± SD, one‐way ANOVA with Tukey's multiple comparisons test). F) Wet to dry (W/D) lung weight ratios (*n* = 6, mean ± SD, one‐way ANOVA with Tukey's multiple comparisons test). G) Total cell count in BALF (*n* = 6, mean ± SD, one‐way ANOVA with Tukey's multiple comparisons test). H) Body weight during the treatment (*n* = 6, mean ± SD, Student's *t*‐test). I) Survival (*n* = 8). * vs. healthy group. # vs. BLM+PBS group. Significance was indicated as *p < 0.05, **p < 0.01, and ***p < 0.001.

Next, we evaluated the antifibrotic efficiency of PAMD@PFOB/siSTAT3 in vivo. The EPs (15 µg siRNA per mouse) were intratracheally instilled on day 14 and continued every 3 d for a total five doses (**Figure** **8**A). Histopathological analysis of lungs from the untreated fibrosis group (BLM+PBS) exhibited loss of normal alveolar appearance and thickening of the alveolar wall (Figure 8B). Increased airway exudate (edema) was also observed. Treatment with PAMD@PFOB/siSTAT3 notably alleviated lung damage, including interstitial edema and thickening of the alveolar wall (Figure 8B). Histopathological scores were assigned to the lung tissue sections in a blinded manner by a pathologist (Figure 8C) using the following scale: 1 = normal lung, 2 = mild inflammation, 3 = moderate inflammation, 4 = obvious inflammation, 5 = severe inflammation. As expected, the untreated (BLM+PBS) group showed severe inflammation and the treatment with PAMD@PFOB/siSTAT3 decreased the pathology score significantly.

Pulmonary collagen deposition was evaluated by Masson's trichrome staining (Figure 8B, blue area) and a hydroxyproline (HYP) assay (Figure 8D). The untreated fibrosis group showed 5.7 times more collagen deposition than healthy mice. Treatment with PAMD@PFOB/siSTAT3 EPs decreased collagen content by ≈50% when compared to untreated controls. This result is consistent with the inhibition of activation and migration of HPLFs and suggests that PAMD@PFOB/siSTAT3 EPs reduce activation and proliferation of fibroblasts and decrease ECM production via combined CXCR4 inhibition and STAT3 silencing. Therefore, combined inhibition of CXCR4 and STAT3 gene silencing is protective in BLM‐induced pulmonary fibrosis as shown by the reduced alveolar wall thickening, fibroblast proliferation and migration, myofibroblast differentiation, and HYP content.

Immunohistochemical (IHC) staining of lung sections and RT‐PCR were performed to measure CXCR4 expression (Figure 8B,E). The lungs of animals treated with the PAMD@PFOB/siSTAT3 showed the lowest levels of CXCR4 expression because of the inhibition of fibroblast differentiating into myofibroblast as well as inhibition of the recruitment of CXCR4‐positive fibrocytes from the bone marrow to the lung. We contend that due to improved lung penetration, EPs had superior ability to inhibit CXCR4 expression in vivo when compared to control polyplexes. Pulmonary edema is an indicator of lung fibrosis ^[^ ^39^ ^]^ and can be evaluated by weighing the wet and dry lungs to obtain the wet/dry (W/D) ratio (Figure 8F). The untreated fibrotic lungs had an increased W/D ratio compared to healthy mice. Strikingly, W/D ratios decreased as a result of EP treatment in comparison to control polyplexes. Consistent with this, the total number of cells in bronchoalveolar lavage fluid (BALF), an indicator of inflammatory cell infiltration was significantly decreased following PAMD@PFOB/siSTAT3 treatment (Figure 8G). The elevated total cell counts in BALF correlate with increased lactate dehydrogenase and cytokine levels.^[^ ^40^ ^]^ We predict that the decreased total cell count observed after treatment with PAMD@PFOB/siSTAT3 is likely to be associated with the decreased levels of the inflammatory cytokines and lactate dehydrogenase. This will be corroborated in our follow‐up studies. Severe loss of body weight was observed in the untreated fibrosis group (BLM+PBS) (Figure 8H), while weight loss in the PAMD@PFOB/siSTAT3 treatment group was less severe.

We next determined whether PAMD@PFOB/siSTAT3 treatment improved survival. PAMD@PFOB/siSTAT3 treatment extended mean survival to 52 d when compared to animals that received no treatment (Figure 8I). H&E and Masson's staining of the lungs from two mice that survived out to 60 d showed that EP treatment clearly attenuated the progression of the pulmonary fibrosis (Figure S11, Supporting Information).

**Figure 9:**
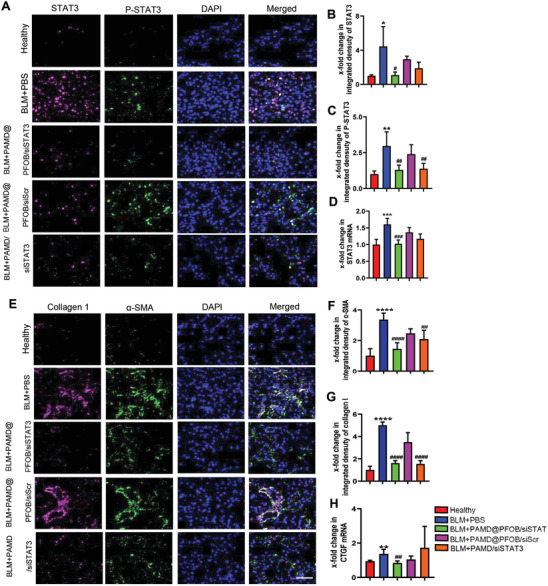
Analysis of the treatment effect on selected biomarkers in pulmonary fibrosis. A) Representative image of immunofluorescence staining for P‐STAT3 (green) and total STAT3 (red) co‐stained with DAPI (staining of nuclei). Scale bar = 100 µm. Quantitative analysis of immunofluorescence staining for B) total STAT3 expression and C) P‐STAT3 expression (*n* = 5, mean ± SD, one‐way ANOVA with Tukey's multiple comparisons test). D) STAT3 mRNA expression (*n* = 6, mean ± SD, one‐way ANOVA with Tukey's multiple comparisons test). E) Representative images of immunofluorescence staining for collagen I (red) and *α*‐SMA (green) co‐stained with DAPI (blue). Scale bar = 100 µm. Quantitative analysis of immunofluorescence staining for F) *α*‐SMA and G) collagen I (*n* = 6, mean ± SD, one‐way ANOVA with Tukey's multiple comparisons test). H) CTGF mRNA expression (*n* = 6, mean ± SD, Student's *t*‐test). * vs healthy group. # vs BLM+PBS group. Significance was indicated as *p < 0.05, **p < 0.01, ***p < 0.001, and ****p < 0.0001.

The effect of the treatments on the p‐STAT3 and total STAT3 expression was assessed by immunofluorescence (IF) staining and RT‐PCR (**Figure** **9**A–D). The untreated group had significantly increased expression of all markers, whereas the PAMD@PFOB/siSTAT3 treatment decreased both the total STAT3 expression and reduced STAT3 activation. As evident from the immunofluorescence staining for collagen I and *α*‐SMA, the PAMD@PFOB/siSTAT3 treatment significantly decreased the overall ECM production (Figure 9E–G). Connective tissue growth factor (CTGF) is a vital growth factor that is related to the tissue repair and pulmonary fibrogenesis.^[^ ^40b^ ^]^ Evaluation of the CTGF mRNA expression in the lung showed significantly elevated CTGF expression in the untreated mice and attenuation of the expression with PAMD@PFOB/siSTAT3 (Figure 9H).

Previously, studies proved that inhibition of STAT3 signaling using pharmacological methods decreases TGF‐*β*‐dependent fibroblast activation and attenuates fibrosis in murine models.^[^ ^26^, ^41^ ^]^ Mice with conditional deletion of STAT3 in fibroblasts are protected from developing skin fibrosis.^[^ ^42^ ^]^ Because of the epithelial damage in IPF, alveolar epithelial cells undergo apoptosis and epithelial–mesenchymal transition to provide mesenchymal cells for the initial repair process.^[^ ^43^ ^]^ Residual fibroblasts in the lung interstitium then start to proliferate and migrate to the injury site, where they boost the ECM deposition.^[^ ^7^ ^]^ Dual targeting of CXCR4 and STAT3 signaling interferes with these crucial steps in the pathology of IPF and is thus a possible therapeutic opportunity.

## 3 Conclusion

In summary, we report EPs capable of safe and effective siRNA delivery to the lungs. The EPs show deep lung penetration and cytosolic siRNA delivery to mediate highly effective pulmonary STAT3 silencing and CXCR4 inhibition in a mouse model of pulmonary fibrosis upon intratracheal administration. The EPs developed in this study: i) bind siRNA, ii) improve cytosolic siRNA delivery, iii) improve lung penetration and pulmonary siRNA delivery, and iv) achieve therapeutic gene silencing in pulmonary fibrosis. Taken together, the reported findings may lead to a development of novel treatment of IPF via noninvasive delivery.

## 4 Experimental Section

### Materials

Fetal bovine serum (FBS), Dulbecco's phosphate buffered saline (PBS), Dulbecco's modified Eagle medium (DMEM), Ham's F‐12 medium, Amphotericin B, trypsin, penicillin, and streptomycin were brought from Thermo Scientific (Waltham, MA). Hexamethylenebisacrylamide (HMBA) and PFOB were purchased from Sigma‐Aldrich. AMD3100 was from Biochempartner (Shanghai, China). All siRNA (siScr, sense strand, 5′‐UUC UCC GAA CGU GUC ACG UTT‐3′; siSTAT3, sense strand for mice, 5′‐GGU CAA AUU UCC UGA GUU GUU‐3′; siSTAT3, sense strand for human, 5′‐GGA GAA GCA UCG UGA GUGA‐3′; carboxyfluorescein (FAM)‐ and Cy3‐labeled siRNA), and real‐time (RT)‐PCR primers were purchased from Sigma‐Aldrich. Curosurf (poractant alfa) was purchased from Chiesi, Inc. (USA). Unless otherwise stated, all other reagents were obtained from Fisher Scientific and used as received.

### Cells and Tissues

Deidentified HPLFs, which were isolated from patients with IPF and from non‐disease control (NDC) patients, were provided through the University of Nebraska Medical Center lung tissue biobank with prior approval from the UNMC IRB. All HPLFs were cultured in DMEM with 10% FBS, 1% amphotericin B, 1% l‐glutamine, penicillin (100 U mL^−1^), and streptomycin (100 µg mL^−1^) at 37 °C in a humidified chamber with 5% CO_2_. MPLFs were isolated from the lungs of mice with BLM‐induced pulmonary fibrosis as described before^[^ ^12b^ ^]^ and cultured at 37 °C with 5% CO_2_ in high‐glucose DMEM with 10% FBS and Pen‐Strep (100 U mL^−1^, 100 µg mL^−1^).

CXCR4, STAT3, col1a1, and col1a2 mRNA levels in the HPLFs and MPLFs were measured by RT‐PCR. Cells were homogenized with TRIzol reagent to isolate total RNA following the protocol. Then the total RNA was converted into cDNA via a High‐Capacity cDNA Transcription kit. The PCR reaction was run on the Rotor‐Gene Q (QIAGEN) using iTaq Universal SYBR Green Supermix.

Deidentified human lung parenchyma slides from NDC and IPF patients were provided by the University of Nebraska Medical Center Lung Transplant Biobank, and the slides were sectioned and analyzed with H&E and Masson's trichrome staining. Immunofluorescence dual‐staining for STAT3 and p‐STAT3, collagen‐1, and *α*‐SMA (smooth muscle actin) was performed, and slides observed using a confocal microscope.

### Preparation and Characterization of PAMD@PFOB/siRNA EPs

PAMD was synthesized as described previously by Michael addition of reacting 1:1 molar ratio of HMBA and AMD3100 and stirred under nitrogen in the dark at 37 °C for 3 d.^[^ ^29b^ ^]^ Excess AMD3100 was added, and the reaction was stirred for another 6 h. The product was collected and dialyzed for 3 d against HCl acidified water (pH 3.0) followed by final dialysis against deionized water and lyophilization. ^1^H‐NMR in D_2_O (Figure S1, Supporting Information) using Varian INOVA (500 MHz) was used to confirm the polymer structure. To prepare fluorescently labeled Cy5‐PAMD, Cy5‐NHS ester was conjugated to PAMD in Na_2_CO_3_/NaHCO_3_ buffer (pH = 8.2) and kept overnight to obtain Cy5‐PAMD. To prepare the PAMD@PFOB nanoemulsion, 20 µL of PFOB was added to 2 mL PAMD (0, 0.25, 0.5, 1, 2 mg mL^−1^) in water and ultrasonicated with a probe‐type ultrasonic processor with a 2 mm diameter (Hielscher, UP200ST) with energy limited to 8000 W under 80% output amplitude setting.

Binding of siRNA to the PAMD@PFOB emulsion was evaluated by agarose gel electrophoresis with 2% agarose gel containing 0.5 µg mL^−1^ of ethidium bromide. PAMD@PFOB/siRNA EPs were formed by adding a different volume of emulsion to an siRNA solution to achieve the desired w/w ratio, incubated at room temperature for 30 min, and run in 0.5× Tris/borate/EDTA buffer for 15 min at 110 mV. The gels were imaged under UV with a KODAK Gel logic 100 imaging system. Hydrodynamic diameter and zeta potential of the EPs were measured by using NanoBrook Omni (Brookhaven Instruments, Holtsville, NY). EPs morphology was analyzed using TEM (Tecnai G2 Spirit, FEI Company, USA) with NanoVan negative staining (Nanoprobes, USA). The colloidal stability of the emulsions in PBS was tested by measuring particle size after incubation at 4, 25, and 39 °C for 1–48 h.

### Stability of EPs in Mucus

A mucus stability assay with naked siRNA, PAMD/siRNA, and PAMD@PFOB/siRNA (w/w = 4) EPs was evaluated via agarose gel electrophoresis. Naked siRNA, PAMD/siRNA, and EPs (w/w = 4, 0.2 µg siRNA) were incubated at 37 °C with 10% mucus (diluted in PBS) obtained from patients with COPD. The samples were collected at predetermined time points. Heparin (80 µg mL^−1^) was used to release siRNA, then analyzed by gel electrophoresis. FRET was also used to measure the mucus and pulmonary surfactant stability of EPs against disassembly. Cy5‐PAMD@PFOB/Cy3‐siRNA (w/w = 4) were prepared using the same methods as described above, and EPs containing 5 µg Cy3‐siRNA were incubated with the 10% mucus obtained from COPD patients (2 mL) or 20% of Curosurf (pulmonary surfactant) for 5 and 24 h at 37 °C. The fluorescence intensity at 667 and 568 nm upon excitation at 550 nm was measured by recording the fluorescence emission spectra according to an earlier report.^[^ ^44^ ^]^

A heparin displacement assay was performed to analyze siRNA release from EPs. PAMD@PFOB/siRNA EPs (w/w = 4) were incubated with different concentrations of a heparin solution for 30 min, then assayed by gel electrophoresis. SYBR‐Safe was used to evaluate the amount of released siRNA. EPs with 0.2 µg siRNA were prepared in HEPES buffer, and 100 µL of each EP solution was distributed in a white 96‐well plate. 3 µL 40× SYBR safe was added to each well and incubated for 10 min in the dark. Fluorescence was measured by a SpectraMax iD3 plate reader microplate reader (Molecular Devices, CA) at 500 nm excitation and 555 nm emission wavelengths. An analogous procedure with free siRNA was used and set as 100%.

To evaluate the PAMD@PFOB/siRNA‐mucus interactions, the EPs (w/w = 4, 5 µg siRNA) were mixed with mucus solution (0.3% or 0.5% mucus and diluted in PBS, 2 mL) and incubated for 5 h at 37 °C. The mixture was centrifuged (300× *g*) for 2 min. Then, the precipitates were washed with PBS and treated with NaOH (5 m, 200 µL) for 10 min, and the fluorescence intensity was measured by SpectraMax iD3 plate reader (Ex = 550 nm, Em = 565 nm).^[^ ^44^ ^]^

Multiple particle tracking was used to evaluate the trajectory of particles in the mucus. Briefly, PAMD@PFOB/siRNA or PAMD/siRNA were mixed with COPD mucus (1 mL) and transferred to 1 mL syringe. After equilibration for 1 h at room temperature, 20 s videos of particle movement and particle trajectories were captured using NanoSight (NS300). ImageJ software (Fuji) was used to convert particle movement into metric displacement in both the X and Y directions. The mean square displacement (MSD) was determined as (*X*_∆t_)^2^ + (*Y*_∆t_)^2^.

### Cytotoxicity of PAMD@PFOB Emulsion in HPLFs and MPFLs

Cytotoxicity of PAMD@PFOB emulsion was tested via a CellTiter‐Blue Cell (CTB) Viability Assay (Promega, WI). Cells were seeded onto 96‐well plates at 6000 cells per well, then, after 12–18 h, the cells were treated with a 100 µL of emulsion with increasing concentrations in culture medium for 24 and 48 h. Then, cell viability was evaluated by the CTB reagent according to the manufacture recommendations. The half‐inhibitory IC_50_ values were calculated from a dose–response analysis in GraphPad Prism.

### Fibroblast Proliferation and Migration

The in vitro cell proliferation assay was evaluated by CTB assay. The HPLFs were seeded in a 96‐well plate (6000 cells per well). After 18 h, the cells were treated with EPs (w/w = 4, siRNA 100 × 10^−9^ m) using serum free medium. The medium was replaced after 4 h with medium containing 10% FBS for another 48 h. Cell viability was then measured using CTB assay according to the manufacturer's recommendations. NDC HPLFs treated with PBS served as a negative control and IPF HPLFs treated with PBS served as a positive control.

The transwell migration assay was conducted as previously described.^[^ ^45^ ^]^ For comparison of migration between NDC and IPF HPLFs, the cells were detached and washed with PBS, then re‐suspended in serum free medium (1 × 10^4^, 2 × 10^4^, 4 × 10^4^, and 6 × 10^4^ cells in 300 µL medium per insert, 8.0 µm pores). 600 µL medium with 10% FBS was added into the lower transwell chamber. After 16 h of incubation, the non‐migrated cells in the top chamber were removed. Migrated cells at the bottom were fixed and stained with 0.2% crystal violet. The migrated cells were observed and counted under EVOS xl microscope.

The effect of PAMD@PFOB/siSTAT3 EPs on the myofibroblast migration was evaluated with IPF HPLFs. Cells were detached and washed with PBS, then resuspended in serum free medium. Cells were pretreated with EPs or polyplexes (without emulsion) for 30 min, and 300 µL of serum‐free medium (4 × 10^4^ cells per insert) was added into the cell culture inserts. 600 µL medium with 10% FBS was added into the lower transwell chamber. After 16 h incubation, the non‐migrated cells in the top chamber were removed. Migrant cells were fixed and stained with 0.2% crystal violet. The migrated cells were observed and counted under EVOS xl imaging system. NDC HPLFs treated with PBS served as a negative control, and IPF HPLFs treated with PBS served as a positive control. Results were interpreted as the percentage of migrated cells relative to NDC HPLFs (*n* = 3).

### Cellular Uptake and Intracellular Tracking

HPLFs and MPLFs were seeded in 12‐well plates and adhered overnight before treatment. Cells were then treated with PAMD/FAM‐siRNA or PAMD@PFOB/FAM‐siRNA (w/w = 4, 100 × 10^−9^ m FAM‐siRNA) for 4 h. Cells were washed with PBS, detached and analyzed by flow cytometry. Subcellular distribution of nanoparticles was observed by confocal microscopy. Cells were seeded in confocal dishes 24 h before the treatment. Cells were then treated with PAMD@PFOB/Cy5.5‐siRNA EPs. After 4 h, cells were washed with PBS, stained with Hoechst 33324, and visualized under confocal microscopy (LSM 710, Zeiss, Jena, Germany). To investigate the endosome siRNA escape, HPLFs (IPF) were treated by Cy3‐PAMD@PFOB/Cy5.5‐siRNA. After 4 h incubation, cells were washed with PBS, stained with LysoView (green) and Hoechst 33324, and visualized with confocal microscopy.

### Pulmonary Distribution of EPs in Mice with Pulmonary Fibrosis

All animal experiment protocols were approved by the University of Nebraska Medical Center Institutional Animal Care and Use Committee. Male C57BL/6 mice were injected intraperitoneally with BLM (twice per week, 4 weeks, 30 U kg^−1^) to induce pulmonary fibrosis. On day 28, mice were intratracheally administered 40 µL of EPs prepared with Cy5‐PAMD and FAM‐siRNA (15 µg siRNA per mouse, w/w = 4). At different times post‐treatment (1, 5, and 24 h after the treatment), whole body fluorescence imaging was conducted prior to ex vivo analysis of the fluorescence in individual organs using Xenogen IVIS 200. Lungs were collected, inflated with O.C.T compound to ensure embedded, cut into frozen sections (10 µm), nuclei stained with DAPI, and then imaged by confocal microscopy.

### Ciliary Beat Frequency

After four weeks of treatment with BLM, C57BL/6J male mice were euthanized, the trachea was extracted and opened lengthwise, then placed in protease solution (1.5 mg mL^−1^) for 18–24 h.^[^ ^46^ ^]^ The tracheas were then discarded, and the solutions were centrifuged (200× *g*) to get the MTECs. Then cells were plated onto an uncoated polystyrene plastic culture dish in MTEC basic media with 10% FBS [MTEC basic media consists of a 1:1 solution of Ham's F‐12 and DMEM supplemented with Pen/Strep (1%), amphotericin B (250 µg mL^−1^), gentamicin (40 mg mL^−1^), and glutamine (4 × 10^−3^ m)]. Due to the differential adherence property of MTECs, after 4 h incubation, the adhered fibroblasts were removed, then washed and counted the MTECs. MTECs were seeded on inserts with Type I collagen‐coated membrane and grown in MTEC supplementary media [consists of basic media supplemented with 0.1% insulin, 0.1% transferrin, 0.1% epidermal growth factor (EGF), 0.4% bovine pituitary extract (BPE), 0.1% cholera toxin (CT), 0.001% retinoic acid (RA), and 5% FBS]. By the sixth day, the cells were cultured at ALI.^[^ ^46^ ^]^ After 14–21 d at ALI, a baseline CBF reading was measured and the cells were treated with 100 µL of EPs or polyplexes (w/w = 4, siRNA = 100 × 10^−9^ m). After 48 h, the beating cilia were observed using an inverted phase‐contrast microscopy, and CBF was calculated using the Sisson Ammons Video Analysis (SAVA) method.^[^ ^47^ ^]^

### Antifibrosis Activity of EPs In Vivo

Pulmonary fibrosis was induced in C57BL/6 mice as stated previously; however, on day 14, mice were randomly assigned to four intratracheal treatment groups: PBS, PAMD/siSTAT3, PAMD@PFOB/siScr, and PAMD@PFOB/siSTAT3 (w/w = 4, 40 µL, 15 µg siRNA/mouse). The mice were administered treatment on days 14, 17, 20, 23, and 26. Untreated C57BL/6 mice served as controls. Mouse body weight was recorded during the entire process. At day 30, BALF from three mice of each group was collected, centrifuged for 10 min at (300× *g*) at 4 °C, the cell pellets were re‐suspended in 200 µL PBS, and total cell number enumerated. All mice were euthanized at day 30 and lung tissues were inflated with 400–800 µL formalin and harvested for the histological and biomedical analyses. The pulmonary edema was evaluated by measuring the wet/dry (W/D) weight ratio. The lungs were excised, washed with PBS, and weighed to acquire the wet weight. Then, lung tissue was dried at 65 °C for 3 d to acquire the dry weight and the W/D weight ratio was calculated. STAT3, CXCR4, and CTGF mRNA levels in the lung were measured by RT‐PCR. Lungs were homogenized with TRIzol reagent to isolate total RNA following the protocol. Then the total RNA was converted into cDNA via a High‐Capacity cDNA Transcription kit. The PCR reaction was run on the Rotor‐Gene Q (QIAGEN) using iTaq Universal SYBR Green Supermix. HYP levels in the lung tissues were measured according to the protocol of the Hydroxyproline Assay Kit (Abcam, USA). A 60‐d survival study was also performed with the same treatment regimens previously stated using eight mice per group.

### Immunohistochemical Analysis

IPF HPLFs were seeded in an eight‐well chambered for 24 h before treatment. The cells were then treated by PAMD@PFOB/siRNA EPs or polyplexes (w/w = 4, 100 × 10^−9^ m siSTAT3) in serum‐free medium. After 4 h incubation, replace with fresh medium containing 10% FBS for another 48 h. Cells were fixed for 15 min, blocked with 5% bovine serum albumin and 0.2% Triton X‐100 for 1 h. The cells were incubated with *α*‐SMA primary antibody (1:200) at 4 °C overnight. Then, the cells were rinsed three times in PBS for 5 min each and incubated with anti‐rabbit IgG AlexaFluor 488 secondary antibody (Thermo Fisher) for 1 h at room temperature. Hoechst 33324 was used to stain nuclei for 15 min and imaged by confocal microscopy. NDC HPLFs treated with PBS served as negative controls and IPF HPLFs treated with PBS served as positive controls.

Mouse lung tissues obtained from the treatment groups were inflated and fixed in 4% paraformaldehyde and then stored in 75% ethanol. Then, the lung tissues were embedded in paraffin and sectioned for histochemical analysis with H&E and Masson's trichrome staining. IHC staining for CXCR4 was conducted in tandem with IF dual‐staining for STAT3 and p‐STAT3, collagen‐1 and *α*‐SMA. Lung slides were deparaffinized and treated with endogenous peroxidase inhibitor, then block slides for 10 min with blocking solution, followed by incubating with primary antibodies at 37 °C for 32 min. The slides were rinsed in IF buffer 3 times for 5 min each, then incubated with the Discovery Cy5 or Cy3 Kit. Slides were also counterstained for 5 min with DAPI and then evaluated using confocal microscopy (LSM 710). IHC images were obtained by the EVOS xl microscope (Thermo, USA).

### Statistical Analysis

Results are presented as mean ± SD. Total sample size (*n*) was given for each experiment as follows: in vitro study (*n* = 3–6); in vivo biodistribution study (*n* = 3); in vivo therapeutic study (*n* = 10); survival study (*n* = 8); H&E staining, Masson's trichrome staining, immunochemistry staining, and immunofluorescence staining (*n* = 3–6). One‐way ANOVA test with Tukey's multiple comparisons test was used to analyze differences among multiple groups followed by a comparison test. Student's *t*‐test was used to analyze the statistical significance between two groups, and differences were assessed to be significant. In all the case, *P*‐value < 0.05 was considered statistically significant, and significance was indicated as *p < 0.05 or ^#^ *p* < 0.05, **p < 0.01 or ^##^ *p* < 0.01, ***p < 0.001 or ^###^ *p* < 0.001, and ****p < 0.0001 or ^####^ *p* < 0.0001. All the statistical analysis was performed with GraphPad Prism 8.

## Conflict of Interest

The authors declare no conflict of interest.

## Supporting information

Supporting Information

## Data Availability

The data that support the findings of this study are available from the corresponding author upon reasonable request.
