# Supplementary material for: Perfluorocarbon Nanoemulsions Enhance Therapeutic siRNA Delivery in the Treatment of Pulmonary Fibrosis
Source: Adv Sci (Weinh). 2022 Jan 7;9(8):2103676. doi: 10.1002/advs.202103676 (PMC8922118; doi:10.1002/advs.202103676)
Supplement: Supplementary file 1 — Supporting Information [file ADVS-9-2103676-s001.pdf]

ADVANCED SCIENCE
Open Access

Supporting Information

for *Adv. Sci.*, DOI: 10.1002/advs.202103676

Perfluorocarbon Nanoemulsions Enhance Therapeutic siRNA Delivery in the Treatment of Pulmonary Fibrosis

*Ling Ding, Siyuan Tang, Weimin Tang, Deanna D. Mosley, Ao Yu, Diptesh Sil, Svetlana Romanova, Kristina L Bailey, Daren L. Knoell, Todd A. Wyatt, David Oupický**

Supplemental materials for

Perfluorocarbon Nanoemulsions Enhance Therapeutic siRNA Delivery in the Treatment of Pulmonary Fibrosis

*Ling Ding, Siyuan Tang, Weimin Tang, Deanna D. Mosley, Ao Yu, Diptesh Sil, Svetlana Romanova, Kristina L Bailey, Daren L. Knoell, Todd A. Wyatt, David Oupický**

L. Ding, S. Tang, W. Tang, A. Yu, D. Sil, S. Romanova, D. Oupický
Center for Drug Delivery and Nanomedicine, Department of Pharmaceutical Sciences, College of Pharmacy, University of Nebraska Medical Center, Omaha NE 68198, USA
E-mail: david.oupicky@unmc.edu

D. D. Mosley, K. L. Bailey, T. A. Wyatt
Department of Internal Medicine, Division of Pulmonary and Critical Care and Sleep, University of Nebraska Medical Center, Omaha NE 68198, USA

D. L. Knoell
Department of Pharmacy Practice and Science, College of Pharmacy, University of Nebraska Medical Center, Omaha, NE 68198, USA

T. A. Wyatt
Department of Environmental, Agricultural and Occupational Health, University of Nebraska Medical Center, Omaha NE 68198, USA
Research Service, Department of Veterans Affairs Omaha-Western Iowa Health Care System, Omaha, NE, 68105, USA

This PDF file includes:

Figure S1 to S11.

Figure S1. Synthesis and ^{1}H-NMR of PAMD.

Figure S2. CXCR4 antagonism of PAMD@PFOB vs. PAMD. (A) CXCR4 receptor redistribution assay in U2OS cells expressing GFP-tagged CXCR4 (green). Scale bar = 100 μm. (B) EC50 values determined from the receptor redistribution assay (n = 3). AMD3100 was used as the positive control.

Figure S3. Cytotoxicity of PAMD@PFOB in the HPLFs (IPF), HPLFs (NDC), and MPLFs (IPF).

Figure S4. (A) Migration of HPLFs (NDC&IPF). (B) x-fold changes in CXCR4 mRNA expression between NDC and IPF. (C) STAT3, col1a1 and col1a2 mRNA expression in HPLFs (NDC) and after treatment with PAMD@PFOB/siSTAT3 in HPLFs (IPF).

Figure S5. Physicochemical characterization of PAMD@PFOB/siRNA EPs. Heparin-induced siRNA release from PAMD@PFOB/siRNA emulsion polyplexes (w/w = 4) with increasing heparin concentration.

Figure S6. Stability of PAMD@PFOB/siRNA within pulmonary surfactant. (A) Fluorescence emission spectra of Cy5-PAMD/Cy3-siRNA, and Cy5-PAMD@PFOB/Cy3-siRNA EPs (excitation at 550 nm) after coincubation with 20% pulmonary surfactant for 0 h and 5 h. (B) Pulmonary surfactant stability assay of PAMD@PFOB/siRNA EPs compared with naked siRNA. (C) Pulmonary surfactant stability assay of PAMD@PFOB/siRNA EPs. PAMD@PFOB/siRNA were incubated with pulmonary surfactant for 24 h, then the agarose gel was run directly without using heparin to release siRNA. (D) Integrity of PAMD@PFOB/siRNA after incubation with pulmonary surfactant.

Figure S7. Representative image of BLM-induced pulmonary fibrosis mice. (A) Schematic illustration. (B) Representative images from the histopathological examination of the lung sections with H&E, Masson's staining, and IHC staining of the CXCR4 in BLM-induced pulmonary fibrosis. 4x: Scale bar = 1000 μm. 20x: scale bar = 200 μm. (C) Activation of the STAT3 signaling in BLM-induced pulmonary fibrosis (female mice).

Figure S8. Accumulation of collagen I and expression of α-SMA in BLM-induced PF male and female mice. Representative image of immunofluorescence staining for collagen I (red) and α-SMA (green) co-stained with DAPI (nuclei) (A: male mice. B: female mice. Scale bar = 100 μm). Quantitative analysis of immunofluorescence staining for collagen I (C) and α-SMA (D).

Figure S9. Biodistribution of the PAMD@PFOB/siRNA EPs (w/w = 4, 40 μL/mouse, 15 μg FAM-siRNA per mouse) at different stages of BLM-induced pulmonary fibrosis. (A) Timeline of the biodistribution study. On day 0, 7, 15, 28, PAMD-Cy5@PFOB/FAM-siRNA were given by intratracheal instillation. Five hours after the instillation, whole-body fluorescence imaging was conducted. Then, the animals were sacrificed, and fluorescence of major organs quantified from ex vivo images. Lungs were collected and embedded for frozen tissue specimens, then stained with DAPI (nuclei). Confocal microscopy was used to image the whole lung slice. (B) Fluorescence in major organs. (C) Whole-body fluorescence after intratracheal administration of EPs. (D) Ex vivo quantification of fluorescence distribution in major organs at different time points. (E) Intra-lung distribution of EPs.

Figure S10. Intra-lung distribution of PAMD/siRNA prepared with Cy5-labeled PAMD (red) and FAM-siRNA (green), nuclei (blue).

Figure S11. Representative images from the histopathological examination of the lung sections with H&E and Masson's staining of the two mice that survived for 60 days following treatment with PAMD@PFOB/siSTAT3.

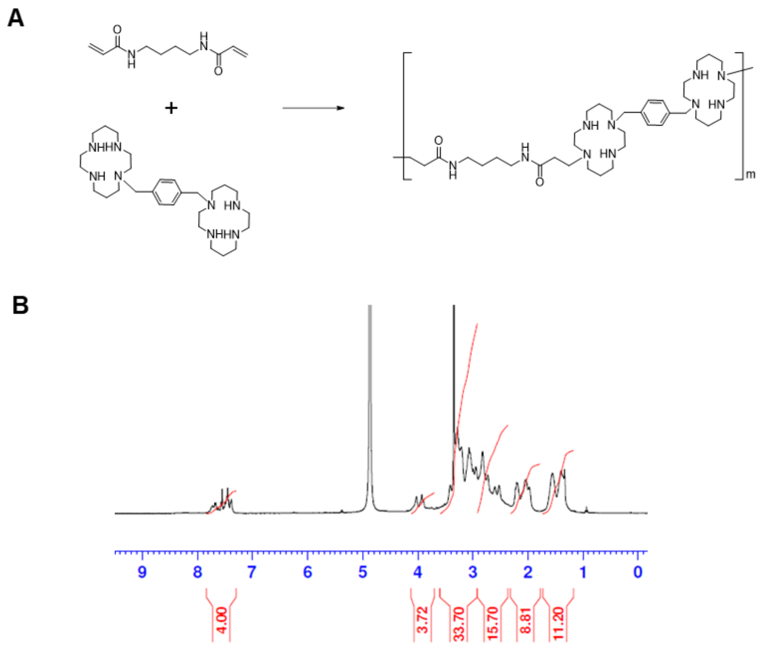

Figure S1. Synthesis and ^{1}H-NMR of PAMD.

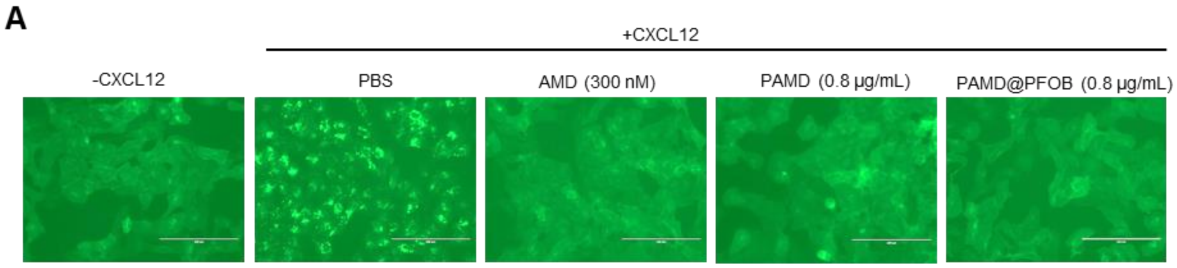

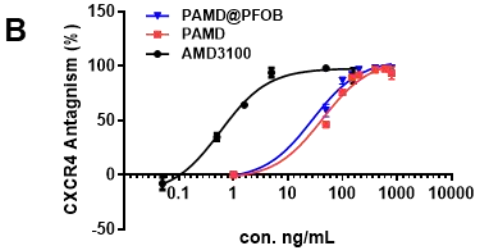

Compound	CXCR4 antagonism (EC50, ng/mL)
AMD3100	0.5816
PAMD	42.71
PAMD@PFOB	28.11

Figure S2. CXCR4 antagonism of PAMD@PFOB vs. PAMD. (A) CXCR4 receptor redistribution assay in U2OS cells expressing GFP-tagged CXCR4 (green). Scale bar = 100 μm. (B) EC50 values determined from the receptor redistribution assay (n = 3). AMD3100 was used as the positive control.

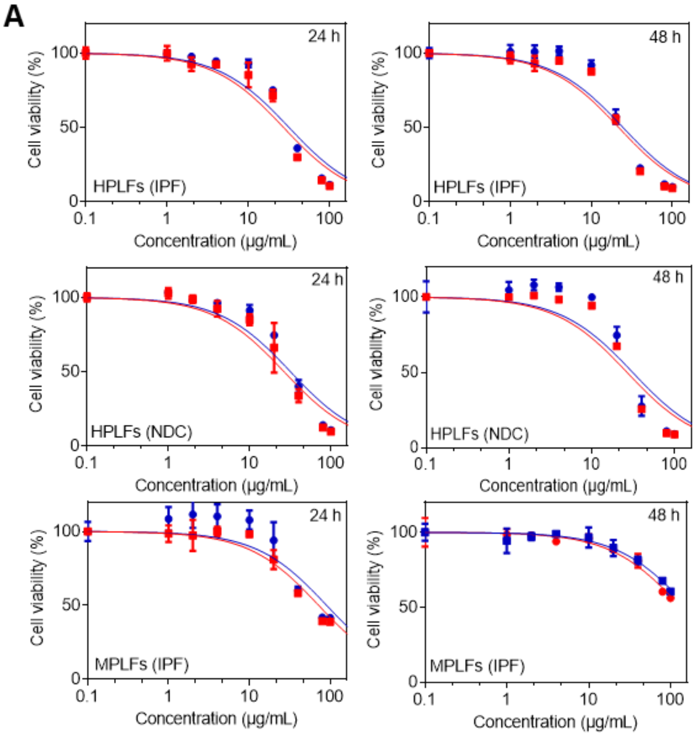

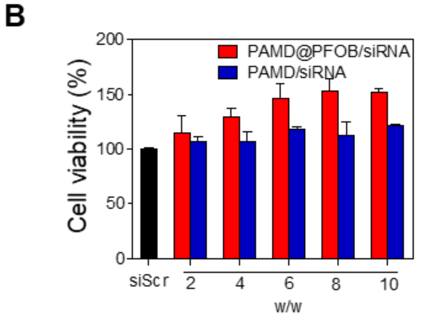

C

IC50 (μg/mL)		PAMD@PFOB	PAMD
HPLFs (IPF)	24 h	31.56	27.02
	48 h	24.53	21.70
HPLFs (NDC)	24 h	31.80	26.51
	48 h	31.21	26.71
MPLFs (IPF)	24 h	90.75	72.39
	48 h	164.2	135.6

Figure S3. Cytotoxicity of PAMD@PFOB in the HPLFs (IPF), HPLFs (NDC), and MPLFs (IPF).

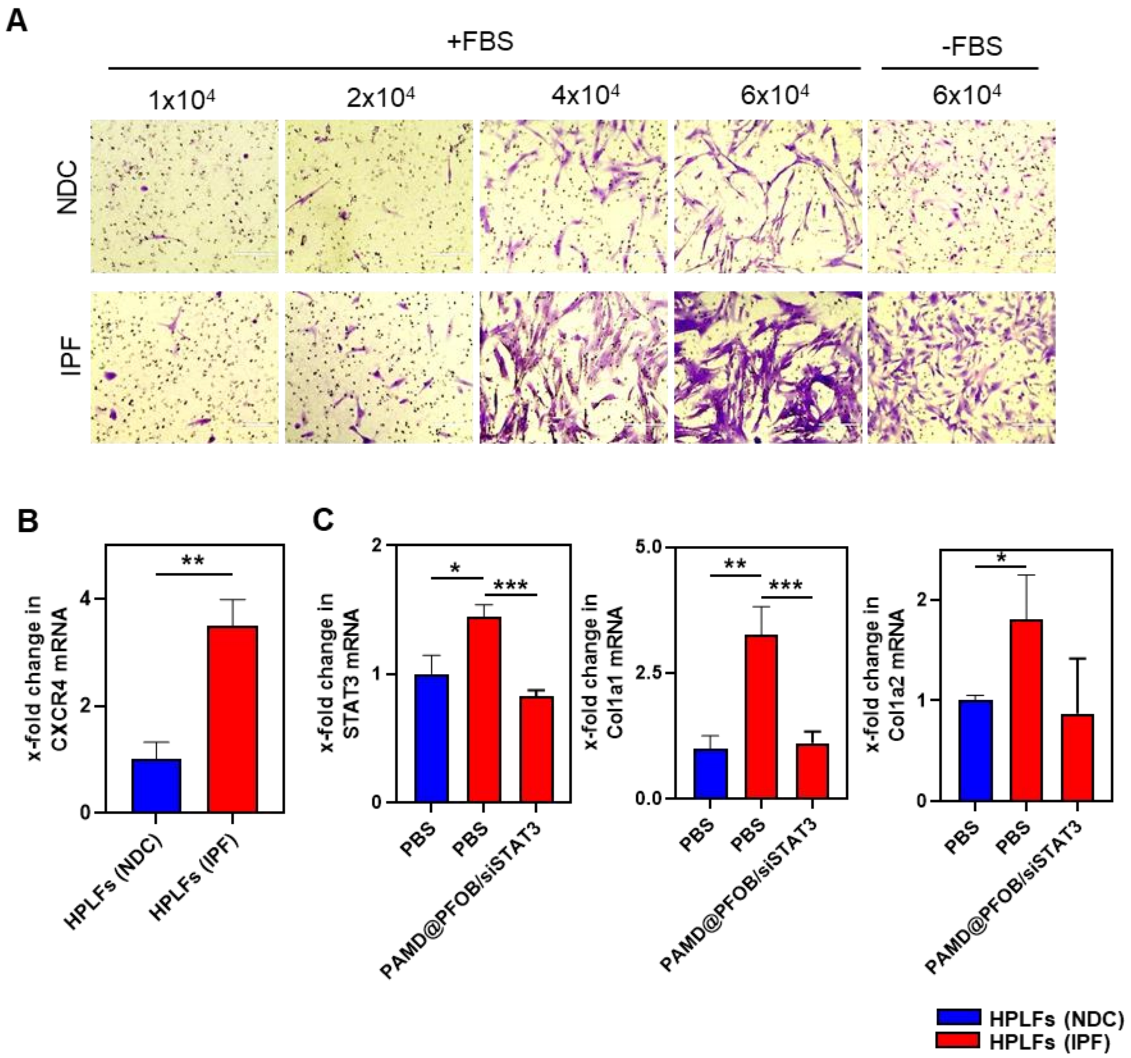

Figure S4. (A) Migration of HPLFs (NDC&IPF). (B) x-fold changes in CXCR4 mRNA expression between NDC and IPF. (C) STAT3, col1a1 and col1a2 mRNA expression in HPLFs (NDC) and after treatment with PAMD@PFOB/siSTAT3 in HPLFs (IPF).

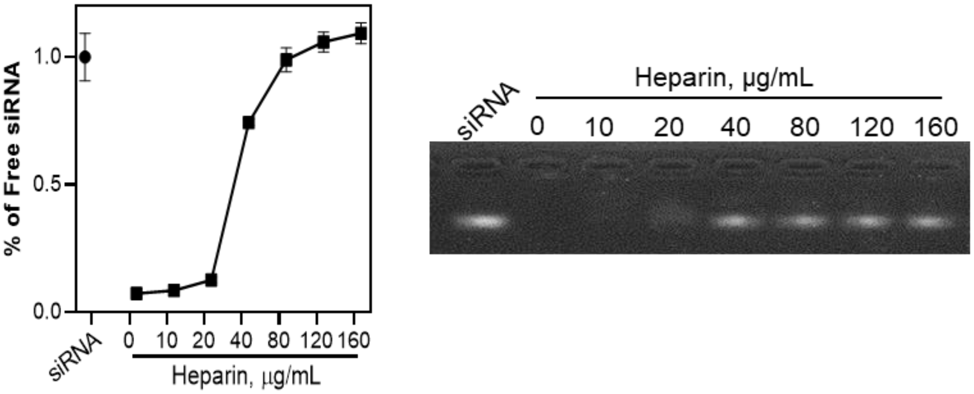

Figure S5. Physicochemical characterization of PAMD@PFOB/siRNA EPs. Heparin-induced siRNA release from PAMD@PFOB/siRNA emulsion polyplexes (w/w = 4) with increasing heparin concentration.

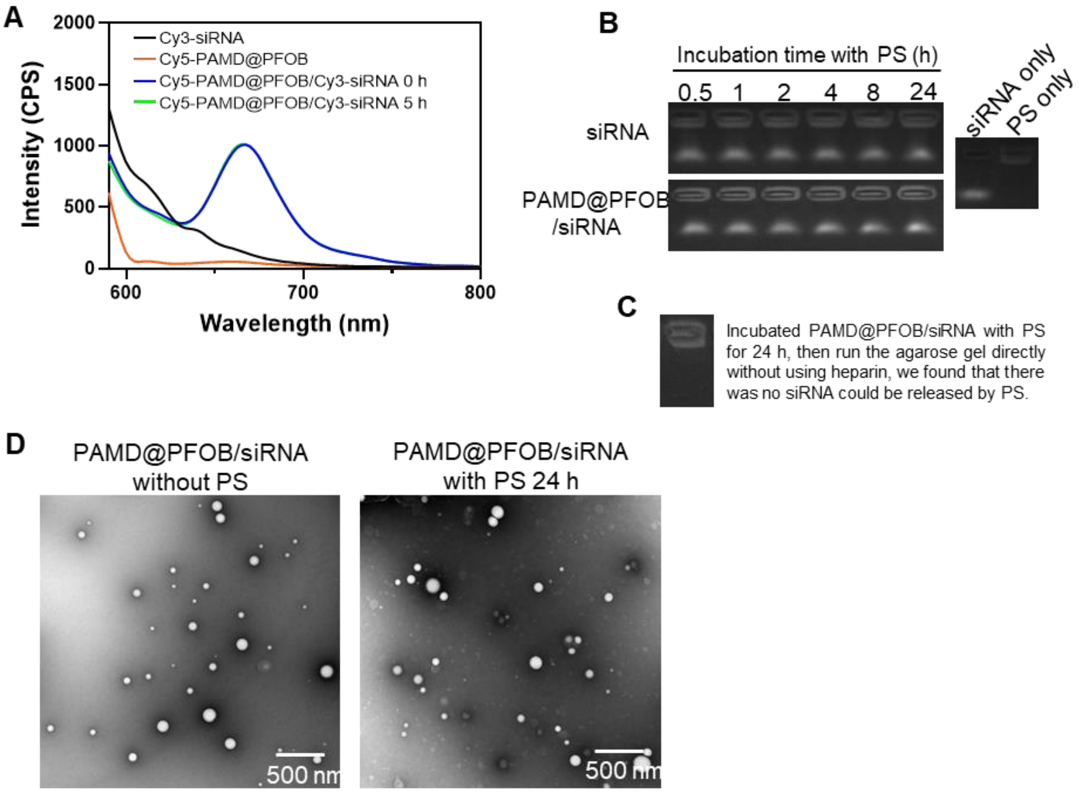

Figure S6. Stability of PAMD@PFOB/siRNA within pulmonary surfactant. (A) Fluorescence emission spectra of Cy5-PAMD/Cy3-siRNA, and Cy5-PAMD@PFOB/Cy3-siRNA EPs (excitation at 550 nm) after coincubation with 20% pulmonary surfactant for 0 h and 5 h. (B) Pulmonary surfactant stability assay of PAMD@PFOB/siRNA EPs compared with naked siRNA. (C) Pulmonary surfactant stability assay of PAMD@PFOB/siRNA EPs. PAMD@PFOB/siRNA were incubated with pulmonary surfactant for 24 h, then the agarose gel was run directly without using heparin to release siRNA. (D) Integrity of PAMD@PFOB/siRNA after incubation with pulmonary surfactant.

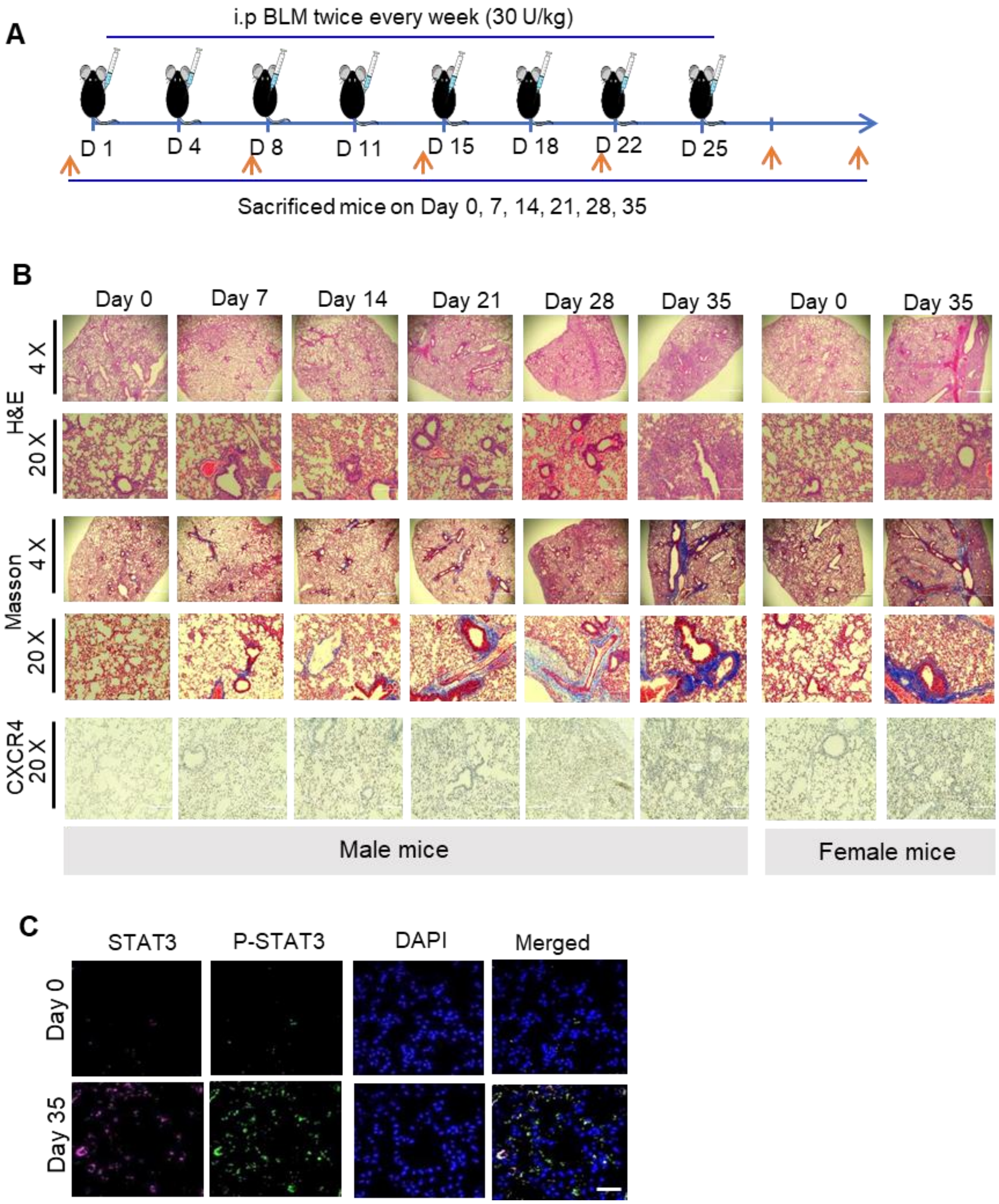

Figure S7. Representative image of BLM-induced pulmonary fibrosis mice. (A) Schematic illustration. (B) Representative images from the histopathological examination of the lung sections with H&E, Masson's staining, and IHC staining of the CXCR4 in BLM-induced pulmonary fibrosis. 4x: Scale bar = 1000 μm. 20x: scale bar = 200 μm. (C) Activation of the STAT3 signaling in BLM-induced pulmonary fibrosis (female mice).

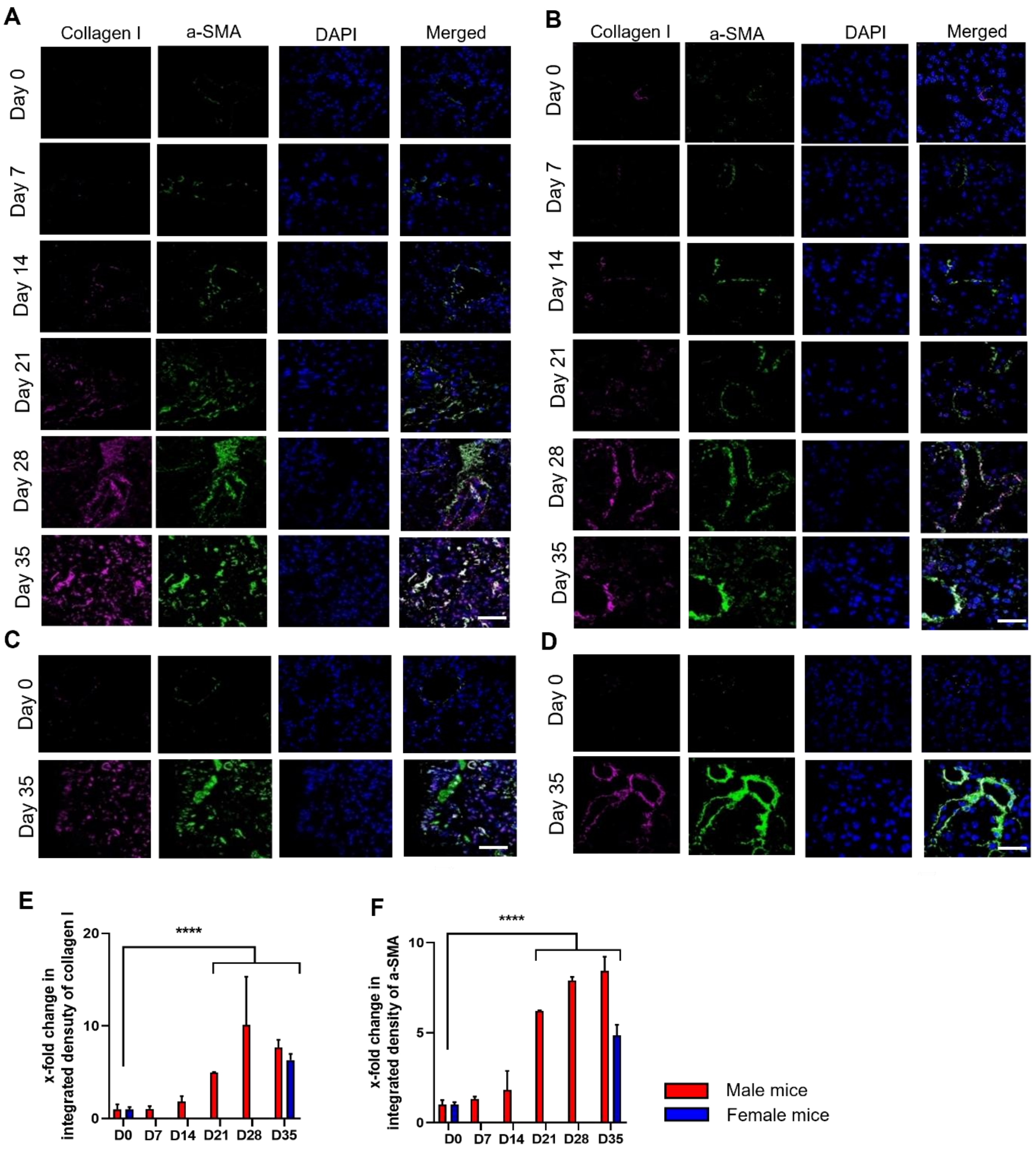

Figure S8. Accumulation of collagen I and expression of α-SMA in BLM-induced IPF male and female mice. Representative image of immunofluorescence staining for collagen I (red) and α-SMA (green) co-stained with DAPI (nuclei) (A&B: male mice. C&D: female mice. A&C: 40x, scale bar = 100 μm. B&D: 63x, scale bar = 60 μm). Quantitative analysis of immunofluorescence staining for collagen I (E) and α-SMA (F).

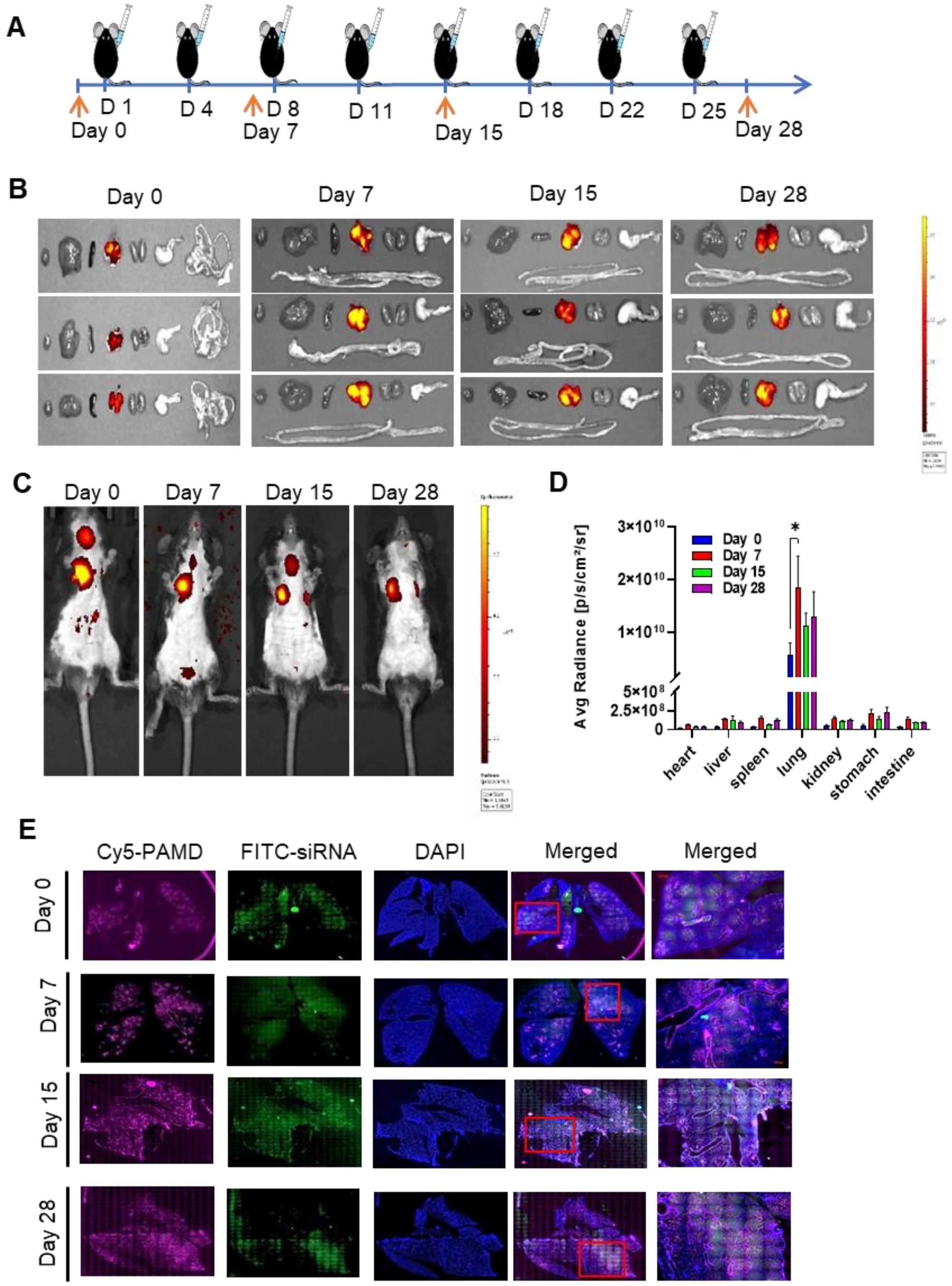

Figure S9. Biodistribution of the PAMD@PFOB/siRNA EPs (w/w = 4, 40 μL/mouse, 15 μg FAM-siRNA per mouse) at different stages of BLM-induced pulmonary fibrosis. (A) Timeline of the biodistribution study. On day 0, 7, 15, 28, PAMD-Cy5@PFOB/FAM-siRNA were given by intratracheal instillation. Five hours after the instillation, whole-body fluorescence imaging was conducted. Then, the animals were sacrificed, and fluorescence of major organs quantified from ex vivo images. Lungs were collected and embedded for frozen tissue specimens, then stained with

DAPI (nuclei). Confocal microscopy was used to image the whole lung slice. (B) Fluorescence in major organs. (C) Whole-body fluorescence after intratracheal administration of EPs. (D) Ex vivo quantification of fluorescence distribution in major organs at different time points. (E) Intra-lung distribution of EPs.

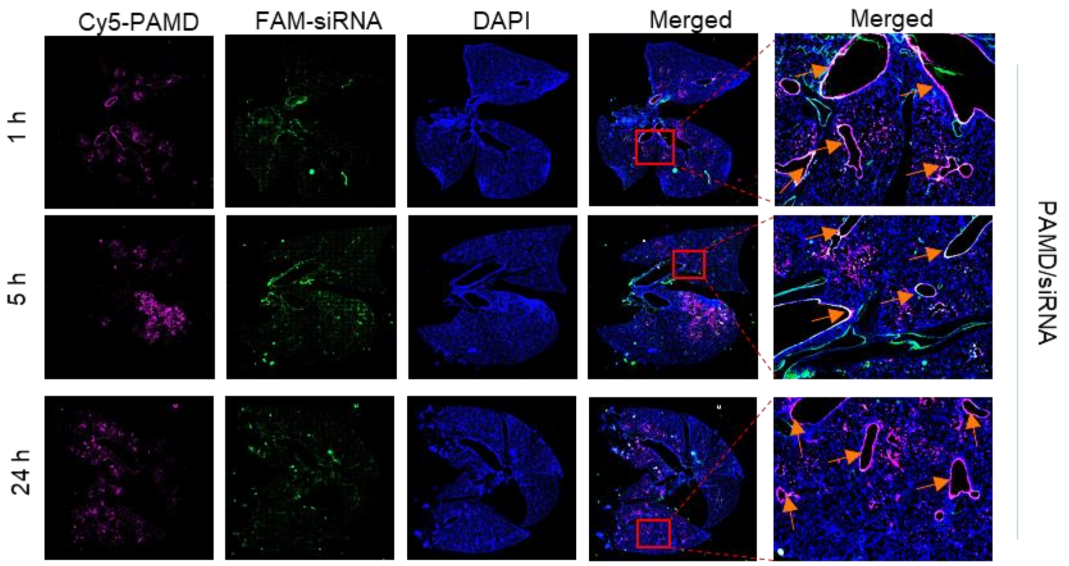

Figure S10. Intra-lung distribution of PAMD/siRNA prepared with Cy5-labeled PAMD (red) and FAM-siRNA (green), nuclei (blue).

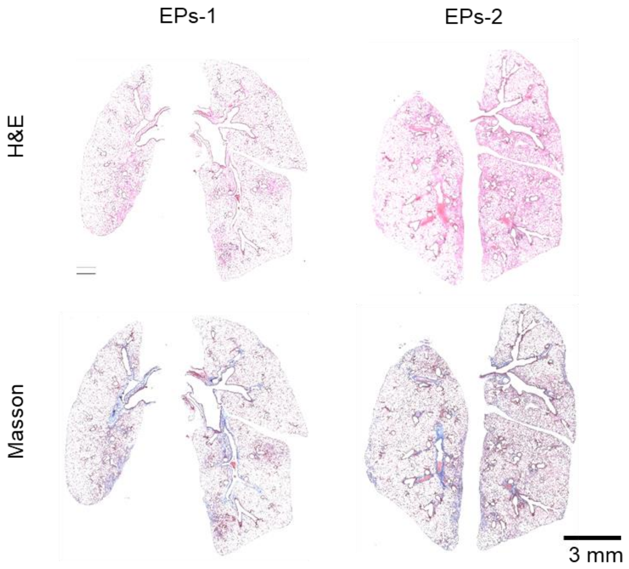

Figure S11. Representative images from the histopathological examination of the lung sections with H&E and Masson's staining of the two mice that survived for 60 days following treatment with PAMD@PFOB/siSTAT3.
